# Influence of plant species, mycorrhizal inoculant, and soil phosphorus level on arbuscular mycorrhizal communities in onion and carrot roots

**DOI:** 10.3389/fpls.2023.1324626

**Published:** 2024-01-15

**Authors:** Umbrin Ilyas, Lindsey J. du Toit, Mehrdad Hajibabaei, Mary Ruth McDonald

**Affiliations:** ^1^ Department of Plant Agriculture, University of Guelph, Guelph, ON, Canada; ^2^ Northwestern Washington Research and Extension Center, Department of Plant Pathology, Washington State University, Mount Vernon, WA, United States; ^3^ Centre for Biodiversity Genomics, Department of Integrative Biology, University of Guelph, Guelph, ON, Canada

**Keywords:** arbuscular mycorrhizal fungi, phosphorus, mycorrhizal inoculants, onion, carrot, plant species, muck soil

## Abstract

Arbuscular mycorrhizal fungi (AMF) are ancient and ecologically important symbionts that colonize plant roots. These symbionts assist in the uptake of water and nutrients, particularly phosphorus, from the soil. This important role has led to the development of AMF inoculants for use as biofertilizers in agriculture. Commercial mycorrhizal inoculants are increasingly popular to produce onion and carrot, but their specific effects on native mycorrhizal communities under field conditions are not known. Furthermore, adequate availability of nutrients in soils, specifically phosphorus, can reduce the diversity and abundance of AMF communities in the roots. The type of crop grown can also influence the composition of AMF communities colonizing the plant roots. This study aimed to investigate how AMF inoculants, soil phosphorus levels, and plant species influence the diversity of AMF communities that colonize the roots of onion and carrot plants. Field trials were conducted on high organic matter (muck) soil in the Holland Marsh, Ontario, Canada. The treatments included AMF-coated seeds (three to five propagules of *Rhizophagus irregularis* per seed) and non-treated onion and carrot seeds grown in soil with low (~46 ppm) and high (~78 ppm) phosphorus levels. The mycorrhizal communities colonizing the onion and carrot roots were identified by Illumina sequencing. Five genera, *Diversispora*, *Claroideoglomus*, *Funneliformis*, *Rhizophagus*, and *Glomus*, were identified in roots of both plant species. AMF communities colonizing carrot roots were more diverse and richer than those colonizing onion roots. *Diversispora* and *Funneliformis* had a 1.3-fold and 2.9-fold greater abundance, respectively, in onion roots compared to carrots. *Claroideoglomus* was 1.4-fold more abundant in carrot roots than in onions. Inoculation with *R. irregularis* increased the abundance and richness of *Rhizophagus* in AMF communities of onion roots but not in carrot roots. The soil phosphorus level had no effect on the richness and diversity of AMF in the roots of either crop. In summary, AMF inoculant and soil phosphorus levels influenced the composition of AMF communities colonizing the roots of onion and carrot plants, but the effects varied between plant species.

## Introduction

Arbuscular mycorrhizal fungi (AMF) are obligate symbionts that colonize plant roots (Smith and Read, 2008). Mycorrhizal associations are thought to have existed for 400 million years and have assisted in land colonization by terrestrial plants (Redecker et al., 2003; Humphreys et al., 2010). Approximately 80% of terrestrial plants are colonized by AMF (Smith and Read, 2008). AMF have a worldwide distribution and are well-adapted to various ecosystems (Dickie et al., 2013; Averill et al., 2014). AMF assist host plants with the absorption of water and essential nutrients, such as phosphorus and nitrogen, from the soil (Augé, 2004; Smith and Read, 2008; Chen et al., 2017; Chandrasekaran et al., 2019). In addition, AMF can improve the tolerance of host plants to biotic and abiotic stresses (Montesinos et al., 1995; Fritz et al., 2006; Pozo and Azcón-Aguilar, 2007; Fiorilli et al., 2009; Campos-Soriano et al., 2012; Sun et al., 2018; Chandrasekaran et al., 2019). AMF belong to the class Glomeromycetes in the phylum Mucoromycota (Spatafora et al., 2016). However, the taxonomic assignment of AMF is still evolving. Initially, AMF were identified based only on morphological characteristics (Morton and Benny, 1990), which did not reflect the full scope of their phylogeny. Molecular studies have revealed the molecular characteristics and phylogenetic relationships among AMF species. Nonetheless, the taxonomic position of some AMF remains uncertain, even at the family level (Schüßler and Walker, 2010). To date, Glomeromycetes consists of five orders (Archaeosporales, Diversisporales, Entrophosporales, Glomerales, and Paraglomerales), 14 families, 29 genera, and approximately 230 species (Morton and Redecker, 2001; Schußler et al., 2001; Sieverding and Oehl, 2006; Walker et al., 2007; Palenzuela et al., 2008; Schüßler and Walker, 2010; Oehl et al., 2011; Spatafora et al., 2016; Błaszkowski et al., 2022).

The discovery of the important role of AMF in the uptake of nutrients by plants has led to the development of AMF inoculants and their use as bio-fertilizers in agriculture (Adholeya et al., 2005; O’Callaghan, 2016). Commercial AMF inoculants are available in liquid formulations, granules, wettable powders, and seed coatings (Adholeya et al., 2005; Rocha et al., 2019). Inoculants can be applied as root dips before sowing, in-furrow at seeding, and planting stock in nurseries (Adholeya et al., 2005; Schwartz et al., 2006). Seeds coated with AMF inoculants are considered a feasible and cost-effective method for large-scale application of inoculants (Ehsanfar and Modarres-Sanavy, 2005; O’Callaghan, 2016). Therefore, AMF inoculants are commonly used as seed coatings by vegetable farmers. In Ontario, Canada, approximately 10% of onion seeds used by growers are coated with AMF inoculants, and almost 90% of onion transplants are treated with AMF (Bridget Visser, Sale representative, Stokes Seeds, Ontario, personal communication). These inoculants mostly contain non-native AMF species belonging to the genera *Gigaspora*, *Funneliformis*, and *Rhizophagus* (Schwartz et al., 2006; Barr, 2010; Berruti et al., 2013). Isolates of *Rhizophagus irregularis* (Błaszk., Wubet, Renker & Buscot) C. Walker & A. Schüßler (2010), in particular, are used extensively as AMF inoculants (Adholeya et al., 2005; Wu et al., 2005; Schwartz et al., 2006; Siddiqui and Akhtar, 2007; Garmendia and Mangas, 2014). *R. irregularis* was previously known as *Glomus irregularis* (Schenck and Smith, 1982) and misidentified as *G. intraradices* in some earlier studies (Stockinger et al., 2009; Walker et al., 2021).

The increase in the use of AMF inoculants in agriculture over the last few decades has raised ecological questions. The introduction of non-native AMF may have negative ecological consequences such as species invasion (Schwartz et al., 2006) and loss of biodiversity (Mckinney and Lockwood, 1999). However, there is a lack of understanding of how non-native AMF inoculants interact with native AMF in plant roots and agricultural soils. To date, there has been no reports of widespread invasion or other ecological problems resulting from the application of non-native AMF inoculants to crops or soil (Schwartz et al., 2006). The problem of species invasion from the use of AMF inoculants in agriculture does not exist, and this effect has not yet been detected (Schwartz et al., 2006). Thus, further research is needed to determine the interactions and other effects of applying non-native AMF inoculants to native AMF. In this study, we hypothesized that the use of AMF inoculants would reduce the diversity of AMF communities colonizing the roots of onion and carrot, two of the main vegetable crops grown in Holland Marsh, Ontario, Canada (OMAFRA, 2017).

Mycorrhizal symbiosis can be negatively affected by the level of nutrients available in soil (Smith and Read, 2008). Soil phosphorus is a primary factor driving the diversity and composition of AMF communities (Gosling et al., 2013; Koorem et al., 2014; Liu et al., 2016). A greater concentration of available phosphorus in the soil can reduce the dependence of plants on AMF for phosphorus uptake, inhibit root colonization by AMF, and negatively affect the diversity of AMF communities colonizing plant roots (Gosling et al., 2006; Liu et al., 2016; du Toit et al., 2019; Qi et al., 2022). The application of phosphorus to soil is a standard practice in the commercial production of onions and carrots in Ontario (OMAFRA, 2017). We hypothesized that high soil phosphorus levels and the application of phosphorus fertilizer would have a negative effect on the composition and diversity of AMF communities colonizing the roots of onion and carrot crops in the Holland Marsh.

AMF are obligate symbionts (Smith and Read, 2008), and the plant species grown can influence the composition of AMF communities colonizing roots (Johnson, 2010; Vályi et al., 2016), even though most AMF species are non-host-specific, as they can colonize a wide range of plant species (Smith and Read, 2008). However, different plant species grown in the same soil can have different AMF communities (Gollotte et al., 2004; Gosling et al., 2013). Host plants were assumed to select the most beneficial AMF species for colonization under specific environmental and nutritional conditions (Walder and van der Heijden, 2015). In this study, we hypothesized that there would be differences in AMF communities colonizing the roots of two different annual vegetable crops, onion and carrot, grown in the same environment. Onion and carrot are commonly grown in the high organic matter (45%–80%) muck soils of the Holland Marsh region near Bradford, Ontario (OMAFRA, 2017). Onion crops are cultivated on ~2,300 ha and carrot crops are cultivated on ~3,300 ha in Ontario (OMAFRA, 2017). To our knowledge, no field-based study has identified the AMF communities colonizing the roots of onion and carrot grown on these muck soils in Ontario. Therefore, the objective of this study was to determine the effects of a commercial AMF inoculant applied to onion and carrot seeds, soil phosphorus levels, and plant species on the composition and diversity of AMF communities colonizing onion and carrot crops on muck soils in Ontario, Canada.

## Materials and methods

### Field trial and sampling

A field trial was conducted on muck soil (pH ~6.9, organic matter ~55.6%) at a site near the University of Guelph, Ontario Crops Research Centre, Bradford, Holland Marsh, Ontario, in 2017 (44°02’39.4”N 79°35’0.37”W). The site had an onion-carrot rotation over several years in both the low- and high-phosphorus plots, as this is a common practice by all the growers in the Holland Marsh, so this was the main plot factor. The site had plots with two levels of pre-plant soil phosphorus: relatively low (~46 ppm) and high (~78 ppm). There were four replicate plots for each phosphorus level. The low-phosphorus plots had no added phosphorus fertilizer since 2010. Phosphorus fertilizer in the form of MicroEssentials SZ (12-40-0-10S-1Zn) was applied at 100 kg phosphate ha^−1^ to plots for high soil phosphorus before seeding. The terms “low phosphorus soils” and “high phosphorus soils” are used in this article for convenience. Other plant nutrients besides phosphorus were broadcast onto all plots at rates recommended by the Ontario Ministry of Agriculture, Food and Rural Affairs (OMAFRA, 2017) in the spring. The onion cv. Trailblazer (Stokes Seed Ltd., Ontario, Canada) was directly seeded (~35 seeds m^−1^ row) with a Stanhay precision seeder on 17 May 2017. Each onion plot was four rows wide × 20 m long, with 40-cm row spacing. The carrot cv. Cellobunch (Stokes Seed Ltd.) was seeded (~65 seeds m^−1^ row) on 04 June 2017. Each carrot plot included two rows × 20 m long, with row spacing of 66 cm. The treatments were seeds coated with an AMF inoculant, and non-treated seeds of the same cultivar and seed lot were planted on the low and high soil phosphorus plots. Commercially available AMF-coated seeds, such as AGTIV Seed Endomycorrhizal Inoculum PTB297, were provided by Premier Tech, Quebec, Canada. The company applied three to five propagules of *R. irregularis* per seed. The onion plots were set up adjacent to but 2 m apart from the carrot plots. The field trial was set up as a randomized complete block design arranged as a split-split plot with four replicate blocks. The main plot treatment was plant species (onion or carrot), the subplot treatment was soil phosphorus level (low or high), and the sub-subplot treatment was AMF (*R. irregularis*) (AMF-coated seeds or non-treated seeds).

AMF colonization was assessed in the fine roots (<2 mm) of onion and carrot plants collected from all replicate plots of each treatment combination. The presence of AMF colonization in the roots was confirmed by observing the roots under a microscope at ×60 magnification. Onion roots were collected at the bulb initiation stage on 31 July, and carrot roots were sampled at the four- to five-leaf stage on 7 August. Fourteen plants were harvested from two rows (seven consecutive plants per row) in each sub-subplot to collect fine roots. The soil attached to the roots was removed by gently shaking them. Fine roots were collected from each plant using forceps, and a composite sample of 2 g of fine roots was prepared for each plot. The roots were then washed with tap water to remove soil particles, cut into 2-cm-long pieces, and stored in 95% isopropanol at room temperature in screw-top glass vials (21 mm × 70 mm, Fisher Brand) until they were used for DNA extraction.

### DNA extraction and PCR assays

The fine roots were dried on autoclaved paper towels in a fume hood to evaporate ethanol. Approximately 50 mg of dried roots from each plot was used to extract DNA. The dried roots were placed in an autoclaved, screw-capped vial (2.5 µL) with a sterilized tungsten bead, immediately frozen in liquid nitrogen, and stored at −80°C for at least 48 h. The frozen roots were then milled into a powder using a Bead Ruptor operated for 1 min at a medium speed. DNA was extracted from milled roots using the DNeasy PowerSoil Kit (QIAGEN, Canada) according to the manufacturer’s instructions, with one modification. The final elution volume of the extracted DNA was reduced from 100 to 30 µL. The quantity of DNA from the root samples ranged from ~19 ng µL^−1^–30 ng µL^−1^. DNA extracted from samples of two commercial AMF inoculants, AGTIV (Premier Tech, Quebec, Canada) and RootRescue (Environmental Products Inc., Ontario, Canada), were used as positive control samples in the PCR assay. The granular form of AGTIV consists of a single AMF species, *R. irregularis*, while RootRescue contains nine AMF species, including *R. irregularis*. Approximately 10 mg of each commercial product was used for DNA extraction using a DNeasy PowerSoil Kit (QIAGEN, Canada) according to the manufacturer’s instructions.

A primer pair, AMV 4.5NF-AMDGR (Sato et al., 2005), was used to amplify the DNA sequences of AMF extracted from onion and carrot roots. Both primers targeted the small subunit of the 18S RNA gene (18S rRNA) (**Supplementary Figure S1**). The primer pair AMV 4.5NF-AMDGR amplified a 577 bp to 834 bp region within the 18S rRNA (**Supplementary Figure S1**) and produced an amplicon of ~280 bp (Sato et al., 2005). The primer pair was barcoded using Illumina adapters.

Polymerase chain reaction (PCR) assays were performed using an Eppendorf thermocycler (MC Pro SepServices). The PCR reaction mixture (total of 20 µL) consisted of 0.5 µL dNTPs (10 mM concentration), 0.5 µL of each primer (10 µM concentration), 2.5 µL 10× PCR buffer without MgCl_2_, 1 µL MgCl_2_ (50 mm concentration), and 0.5 µL Invitrogen Platinum Taq DNA Polymerase (Fisher Scientific, Canada). The PCR conditions were as described by van Geel et al. (2015) with some modifications to the cycling conditions. The PCR conditions for the primer pair AMV 4.5NF-AMDGR were optimized by initial denaturation for 2 min at 94°C, followed by 25 cycles of 45 s at 94°C, 45 s at 60°C, and 45 s at 72°C, followed by a 10-minute final elongation at 72°C. The amplified DNA was separated on a 1.5% agarose gel by electrophoresis to confirm the successful amplification of DNA. The amplified DNA was purified using a MiniElute PCR purification kit (QIAGEN Group, Germany), and the purified DNA was quantified using a fluorimeter (Turner BioSystem Inc., Canada) and sequenced with Illumina MiSeq (Serial MUD828, Experience Genetic Energy) at the Department of Integrative Biology, University of Guelph, Ontario, Canada.

### Illumina MiSeq data analysis

The raw reads were obtained for all the carrot and onion samples from Illumina DNA sequencing, and analyzed via the 18S meta-barcode pipeline available on GitHub at https://github.com/terrimporter/SCVUS_18S_metabarcode_pipeline. The raw reads were obtained as FASTQ files. The reverse and forward reads obtained with the AMV 4.5NF-AMDGR primer pair were paired from end to end using the SEQPREP program (https://github.com/jstjohn/SeqPrep). The quality score threshold for maintaining all reads was set to Q30. The paired reads were filtered to a quality of Phred 20 at the ends of the reads, for a minimum overlap of 25 bp.

The primer were trimmed from paired and unpaired reads using CUTADAPT v 1.13 (Martin, 2011). The trimmed reads were de-replicated with VSEARCH v 2.5.0 (Rognes et al., 2016). The reads were denoised using the USEARCH v. 10.0.240 to remove putative sequencing errors, chimeric sequences, and low-frequency reads including singletons and doubletons. The denoised unique sequences were aligned and clustered to exact sequence variants (ESVs) at 100% similarity using USEARCH v. 10.0.240 and the UNOISE 3 algorithm (Edgar, 2016). Taxa were assigned to ESVs using the SILVA database via the RDP Classifier (Wang et al., 2007). A confidence threshold of ≥90 at the genus level was selected to ensure 90% correct assignment of AMF genera using the reference database.

### Statistical analysis

ESV data were sorted into three sets: plant species, AMF inoculant, and soil phosphorus levels. Each ESV dataset was analyzed at the genus level for AMF community diversity using vegan packages in R v.3.5.1. The data were normalized to mask biases by rarefying the 15th percentile before analysis (Weiss et al., 2017) using the Rrarefy function. Moreover, ESVs <2 for each sample were excluded from the analysis (Egan, 2017).

Variations in the composition of AMF communities associated with plant species, AMF inoculant, and soil phosphorus level were calculated using PERMANOVA via Adonis. PERMANOVA was calculated based on the Bray–Curtis distance matrix with 999 permutations (Kennedy et al., 2018). Multivariate dispersion was calculated using the betadisper() and permutest() functions (999 permutations; alpha = 0.05) via the permute function to validate the PERMANOVA results. A significant PERMANOVA result and insignificant dispersion indicated that the observed differences in the community were due to differences in community composition rather than uneven variation in the data. When both PERMANOVA and dispersion were significant in a dataset, the results were interpreted with caution and more weight was given to the separation of ellipses in Principal Component Analysis (PCA) plots. PCA was used to plot the variation in groups in the multivariate space using the package ape. The differential abundance of ESVs between the two AMF communities for each paired comparison was determined using the Wilcoxon rank-sum test using the package indicispecies. A multiple test correction, with a family wise error rate and Benjamini–Hochberg correction, was applied to the Wilcoxon rank-sum test and computed *q*-values. A logarithmic abundance of ESVs with a value of *q ≤*0.05 was considered significantly different between the two groups. However, some variations in the relative abundance of AMF communities at a value of *q* ~ 0.1 were considered as an important effect of the treatment.

The richness of the ESVs was calculated for non-normalized data using the SpecNumber function in vegan. Richness was determined using non-normalized data, based on the absence or presence of AMF taxa. Two diversity indices, Shannon’s index and Inverse Simpson’s index of diversity, were calculated for the normalized data using vegan. The significance of the richness and diversity indices was analyzed using a split-split-plot design with crop as the main plot, phosphorus as the sub-plot treatment, and AMF inoculant as the sub-sub-plot treatment, using Tukey’s test in the Proc GLIMMIX procedure in SAS. There was no significant three-way interaction between crop, AMF inoculant, and soil phosphorus levels; therefore, significant two-way interactions were used to demonstrate the effect of treatments on the results. All statistical variations in the data were considered significant at *P* ≤0.05. However, some variations were considered an important effect of the treatment on the composition of AMF communities at *P* ≤0.1, depending on the research question.

## Results

In this study, 1,532,940 unique DNA sequences from the roots of onion and carrot plants were assigned to fungal taxa. Approximately 68% of the unique sequences were Mucoromycota, 99% of which belonged to Glomeromycetes. Overall, 468 ESVs belonging to AMF were identified using the AMV 4.5NF-AMDGR primer pair at the genus level with confidence threshold values ≥90.

Taxonomic assignment of ESVs with confidence threshold values >90 revealed that AMF communities colonizing the roots of carrot and onion crops belonged to Diversiporales, Glomerales, and Entrophosporales (**Supplementary Table S1**). Approximately 94% of the identified ESVs of AMF from onion roots and 79% of those from carrot roots belonged to Glomerales. Three families of AMF (Diversisporaceae, Claroideoglomeraceae, and Glomeraceae) were identified in AMF communities colonizing the roots of both crops. Most of the ESVs could be identified to the genus level, but a few ESVs belonging to Glomeraceae were identified only at the family level. At present, there are no Glomeraceae reference genomes that can help identify these ESVs in the genus. Instead, these ESVs were grouped as “Glomerales undefined.” Five AMF genera with confidence threshold values >90 were identified in the AMF communities colonizing the roots of onions and carrots (**Supplementary Table S2**). The identified AMF species had confidence thresholds <90, therefore these were termed as “potential species.” Eleven potential AMF species were identified the onions, and nine in the carrots (**Supplementary Table S2**). Two potential species, namely *Glomus* sp. NBR31 and *Glomus macrocarpum* were identified only in the onion roots (**Supplementary Table S2**).

### AMF communities colonizing roots based on plant species

**Figure 1:**
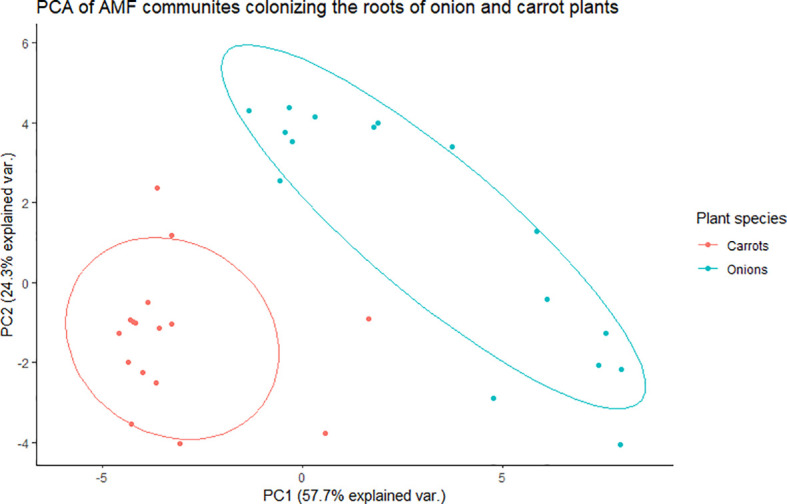
Principal component analysis (PCA) for variation in arbuscular mycorrhizal fungal (AMF) communities colonizing the roots of onion and carrot plants grown in muck soil in a field trial in Holland Marsh, Ontario, Canada. Values in the X and Y axes indicate percentage variation in AMF communities contributed by the first principal component (PC1) and second principal component (PC2) using the Vegan package of R.

**Figure 2:**
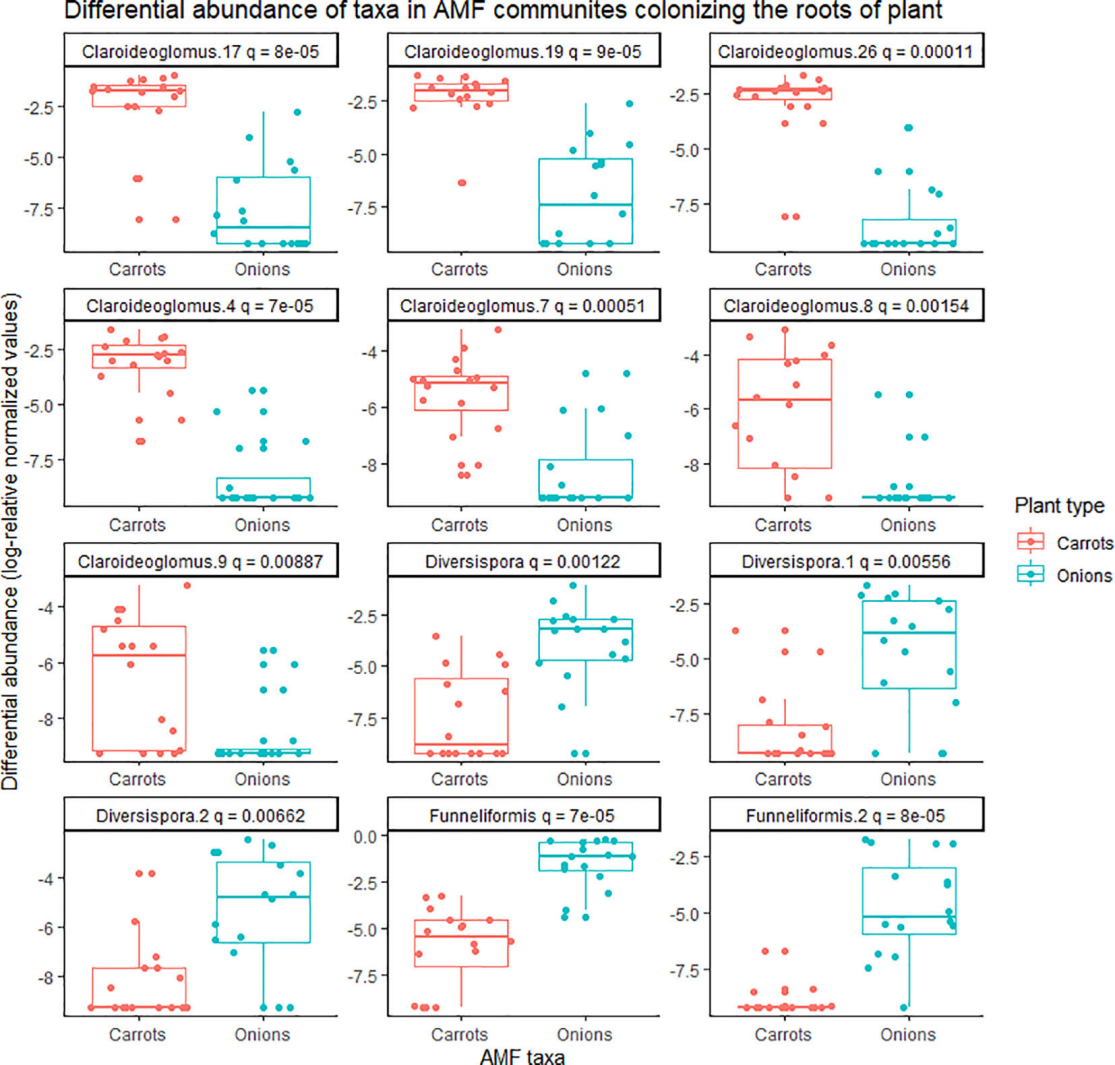
Differential abundance of taxa belonging to arbuscular mycorrhizal fungal (AMF) communities colonizing the roots of onion and carrot plants grown in muck soil in a field trial in Holland Marsh, Ontario, Canada. Only taxa with significant differences (q-value ≤0.05) in relative abundance based on the Wilcoxon rank-sum test are shown. Each taxon represents an ESV with taxonomic assignment at the genus level.

There was a significant effect of plant species (onion vs. carrot) on the composition of AMF communities colonizing roots, based on the PREMANOVA analysis (*P* = 0.001, **Table 1**). Multivariate dispersion was significant between the onions and carrots (**Table 1**). The PCA plot showed two distinct communities in the multidimensional space of AMF colonizing the two crops (**Figure 1**). Since the PCA showed distinct plots, the AMF community structure in the roots of onions was considered different from that in carrots despite the significant dispersion. The PCA plot explained a total of 82% the variance in AMF communities, in which principal component 1 (PC1) represented 57.7% and principal component 2 (PC2) represented 24.3%. Plant species had a significant effect on the diversity of AMF communities (**Table 2**), as the richness of the AMF communities colonizing the roots of carrot was 22% greater than that of AMF communities colonizing onion roots (**Table 2**). Both Shannon’s and Inverse Simpson’s diversity indices were greater (almost double) for carrot roots than for onion roots (**Table 2**). Taxa related to the genera *Rhizophagus*, *Funneliformis*, and *Diversispora* were dominant in the AMF community colonizing the onion roots (**Supplementary Figure S2**). Taxa related to the genera *Claroideoglomus* and *Rhizophagus* were dominant in the carrot roots (**Supplementary Figure S2**). Twelve taxa differed significantly between the two crops (**Figure 2**). Three taxa belonging to the genus *Diversispora* and two taxa belonging to the genus *Funneliformis* were significantly more abundant in the onion AMF communities than in carrot roots, but seven ESVs belonging to *Claroideoglomus* were significantly more abundant in carrot roots than in onion roots (**Figure 2**).

**Table 1:** Permutational multivariate analysis of variance (PERMANOVA) and multivariate dispersion table for arbuscular mycorrhizal fungi colonizing the roots of onions and carrots grown on muck soil in a field trial at Holland Marsh, Ontario.

PERMANOVA^1^				
Source of variation	Df	Sum of Square	Pseudo-*F*	*P-*value
Crop type	1	2.79	15.34	**0.001**
Residual	30	5.47	0.66	
Total	31	8.26		
Application of AMF inoculant to onion seeds	1	0.97	5.04	**0.003**
Residual	14	2.69	0.73	
Total	15	3.66		
Application of AMF inoculant to carrot seeds	1	0.11	87.9	0.505
Residual	14	1.88	0.94	
Total	15	2.00		
Soil phosphorus level in onion	1	0.40	1.72	0.107
Residual	14	3.26	0.89	
Total	15	3.66		
Soil phosphorus level in carrot	1	0.55	5.23	**0.001**
Residual	14	1.47	0.72	
Total	15	2.02		
Multivariate dispersions^2^				
Onions vs. carrots	1	0.15	6.34	**0.016**
Residual	30	0.72		
AMF-coated vs. untreated onion seeds	1	0.00005	0.0094	0.932
Residual	30	0.16179		
AMF-coated vs. untreated carrot seeds	1	0.00351	0.1544	0.679
Residual	28	0.6359		
Low- vs. high-soil P^3^ in onions	1	0.001633	0.8177	0.3731
Residual	30	0.059907		
Low- vs. high-soil P in carrots	1	0.02444	0.8434	0.367
Residual	28	0.81145		

^1^The significance of multivariate analysis of variance was assessed using PERMANOVA with adonis() function (iterations = 999 permutations). The P-value ≤0.05 was considered as significant.

^2^Multivariate dispersion was tested using the betadisper() and permutest() functions (iterations = 999 permutations; alpha = 0.05) revealing a significant homogeneity of group dispersions. The P-value ≤0.05 was considered as significant.

^3^~46 ppm pre-plant soil phosphorus level, and ~78 ppm pre-plant soil phosphorus level.

Bold letters are representing the significant P- values.

**Table 2:** Richness, Shannon`s index of diversity, and the Inverse Simpson’s index of diversity of arbuscular mycorrhizal fungi (AMF) communities colonizing the roots of onion and carrot plants grown on high organic matter (muck) soil in a field trial at Holland Marsh, Ontario, Canada.

Variation	Group	Richness^1^	Shannon’s Index^2^	Inverse Simpson’s index^2^
Plant species	Onion	13 b^3^	1.4 b	3.62 b
	Carrot	17 a	2.0 a	6.17 a
	*P-*value	**0.0043**	**0.0001**	**0.001**
Mycorrhizal Inoculant*crop	AMF-coated onion seeds^4^	18 a	1.95 a	5.36 a
	Untreated onion seeds	7 b	0.9 b	1.89 b
	AMF-coated carrot seeds	16 a	1.95 a	5.78 a
	Untreated carrot seeds	17 a	2.15 a	6.56 a
	*P-*value	**0.0009**	**0.0003**	**0.0184**
Soil phosphorus levels*crop	Onion grown on low-soil P^5^	12 b	1.51 ns	4.34 ns
	Onion grown on high- soil P^6^	14 b	1.34	2.91
	Carrot grown on low-soil P	18 a	2.05	6.62
	Carrot grown on high- soil P	16 ab	2.00	5.71
	*P-*value	**0.0466**	0.5019	0.5781

^1^Richness of the exact variant sequences (ESVs) was calculated from non-normalized data.

^2^ Shannon’s index and Inverse Simpson’s index of diversity were calculated using normalized data.

^3^ Means in a column followed by the same letter do not differ based on Tukey`s test at P ≤ 0.05.

^4^ Commercially available AMF-coated seeded (~3 to 5 propagules of Rhizophagus irregularis per seed) provided by Premier Tech, Quebec, Canada.

^5^~46 ppm pre-plant soil phosphorus level.

^6^~78 ppm pre-plant soil phosphorus level.

Bold letters are representing the significant P- values.

### Effect of mycorrhizal inoculant applied to seed on AMF communities colonizing roots

**Figure 3:**
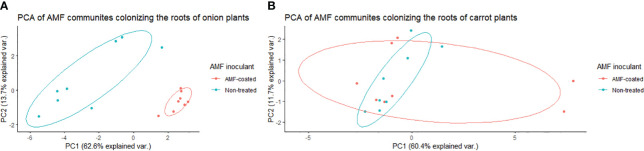
Principal component analysis (PCA) for variation in arbuscular mycorrhizal fungal (AMF) communities colonizing the roots of onion **(A)** and carrot **(B)** plants grown from AMF-coated and untreated seeds in a muck soil in a field trial in Holland Marsh, Ontario, Canada. Values in the X and Y axes indicate percentage variation in AMF communities contributed by first principal component (PC1) and second principal component (PC2) in by using the Vegan package of R.

**Figure 4:**
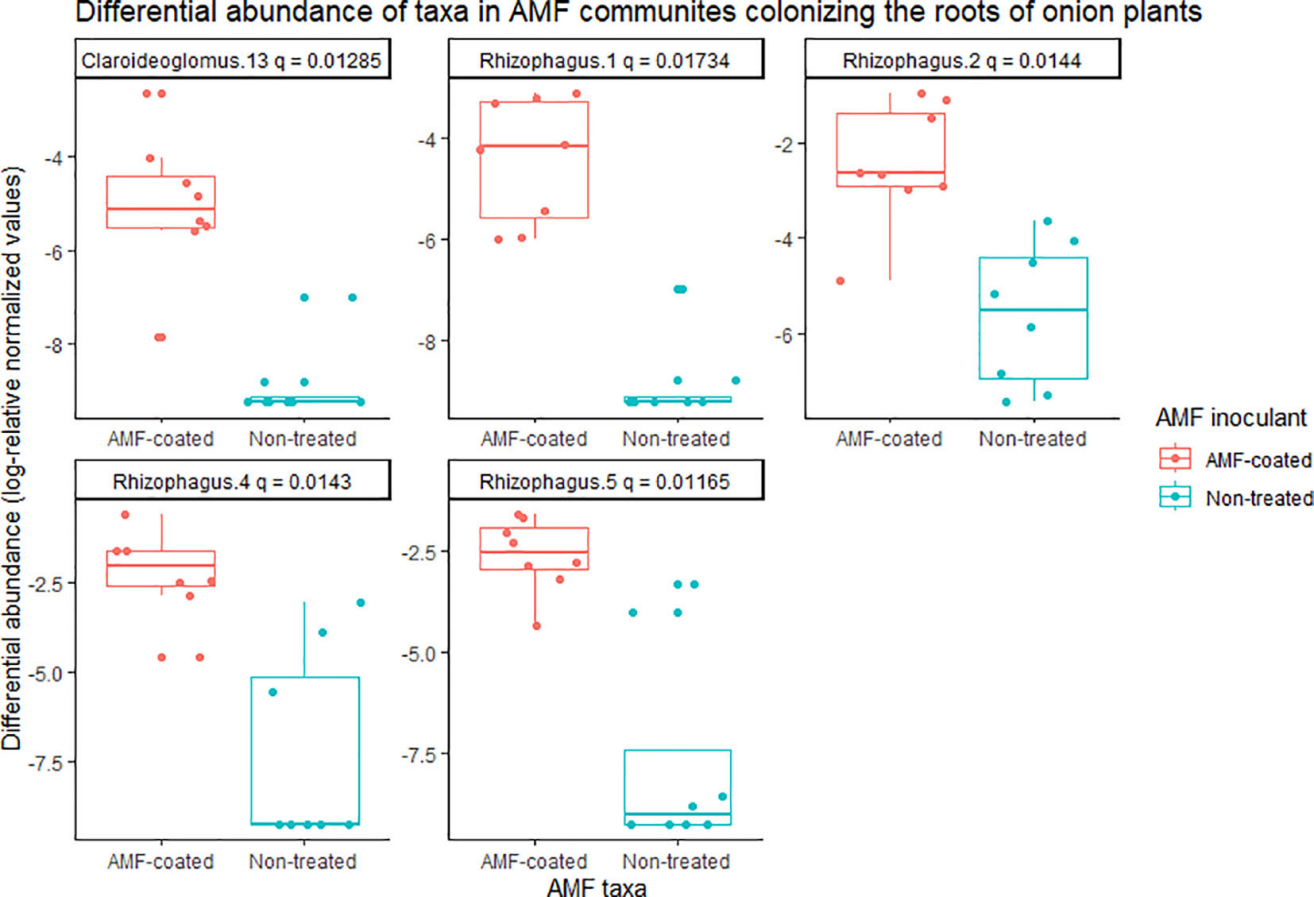
Differential abundance of arbuscular mycorrhizal fungal (AMF) communities colonizing the roots of onion plants grown from AMF-coated and untreated seeds in muck soil in a field trial in Holland Marsh, Ontario, Canada. Taxa with significant differences (q-value ≤0.05) in relative abundance based on the Wilcoxon rank-sum test are shown. Each taxon represents an ESV, along with taxonomic assignment to the genus level.

In onions, there was a significant effect of *R. irregularis* AMF inoculant as a seed coating on the composition of AMF communities colonizing the roots, based on the PERMANOVA (*P* = 0.003), and multivariate dispersion within the groups (*P*= 0.932) (**Table 1**). PCA (A) showed two distinct AMF communities in onion plants grown from AMF-coated seeds compared to the communities in onions grown from non-treated seeds (**Figure 3**). The PCA plot (A) explained a total of 76.3% of the variance in AMF communities, with PC1 representing 62.6% and PC2 representing a variance of 13.7% (**Figure 3**). There was no three-way interaction of crop ∗ AMF-inoculant ∗ soil phosphorus levels for the richness and diversity indices (**Supplementary Table S3**). There was a significant two-way interaction of crop ∗ AMF-inoculant for richness and diversity indices (**Supplementary Table S3**), and this interaction was used to describe the effect of AMF inoculant on AMF communities colonizing the roots. The AMF communities colonizing onions grown from AMF-coated seeds had greater richness, Shannon’s index and Inverse Simpson’s index compared to non-treated seeds (**Table 2**). The increase in the richness of AMF communities of onions from AMF-coated seeds was unexpected, given that the inoculant consisted of a single AMF species, *R. irregularis*. The relative abundance of four taxa belonging to *Rhizophagus* and one taxon belong to *Claroideoglomus* was significantly greater in onions grown from AMF-coated seeds than in those grown from non-treated seeds (**Figure 4**).

In carrots, there was no significant effect on the composition of AMF communities colonizing the roots, based on the PERMANOVA (*P* = 0.505) and multivariate dispersion within the groups (*P*= 0.679) (**Table 1**). PCA (B) showed different but overlapping plots for the variation in AMF communities between carrots grown from AMF-treated and non-treated seeds (**Figure 3**). The PCA plot (B) explained a total of 72% variance in AMF communities, in which PC1 represented 60.4% and PC2 represented a variance of 11.7% (**Figure 3**). There was a greater spread of AMF communities colonizing carrot roots grown from AMF-coated seeds in the PCA plot (B) (**Figure 3**). However, this elliptical spread was primarily driven by two extreme values taht were identified as outliers (**Figure 3B**). Furthermore, the treatment had no significant effect on diversity based on either diversity indices (**Table 2**) or differential abundance of taxa (data not shown).

### Effect of soil phosphorus levels on AMF communities colonizing plant roots

**Figure 5:**
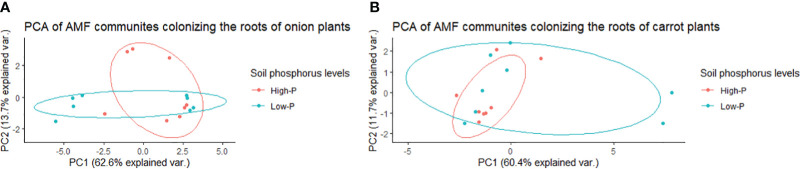
Principal component analysis (PCA) for variation in arbuscular mycorrhizal fungal (AMF) communities colonizing the roots of onion **(A)** and carrot **(B)** plants grown in muck soil with low- and high-phosphorus levels in a field trial in Holland Marsh, Ontario, Canada. Values in the X and Y axes indicate percentage variation in AMF communities contributed by the first principal component (PC1) and second principal component (PC2) using the Vegan package of R. The soil pre-plant phosphorus levels were ~46 ppm for the low phosphorus treatment and ~78 ppm for the high phosphorus treatment.

In onion, there was no significant effect of the two soil phosphorus levels on the composition of AMF communities colonizing roots, based on the PERMANOVA (*P* = 0.107) and multivariate dispersion within the groups (*P* = 0.373) (**Table 1**). The PCA plot (A) showed two distinct plots of AMF communities in the roots of onion plants grown in soil with high vs. low phosphorus, with some overlap (**Figure 5**). The PCA plot (A) explained a total of 76.3% of the variance in AMF communities, in which PC1 represented 62.6% and PC2 represented a variance of 13.7% (**Figure 5**). There was no three-way interaction of crop ∗ AMF-inoculant ∗ soil phosphorus levels for the richness and diversity indices (**Supplementary Table S3**). There was a significant two-way interaction between crop ∗ soil phosphorus levels for richness (**Supplementary Table S3**), and this interaction was used to describe the effect of soil phosphorus on AMF communities colonizing the roots. There were no significant differences in mean richness, Shannon’s and Inverse Simpson’s diversity indices of AMF communities detected in the roots of onion plants growing on low- vs. high-phosphorus soils (**Table 2**). None of the AMF taxa had significantly different relative abundances in the AMF communities colonizing the roots of onions grown in low- and high-phosphorus soils (results not shown).

**Figure 6:**
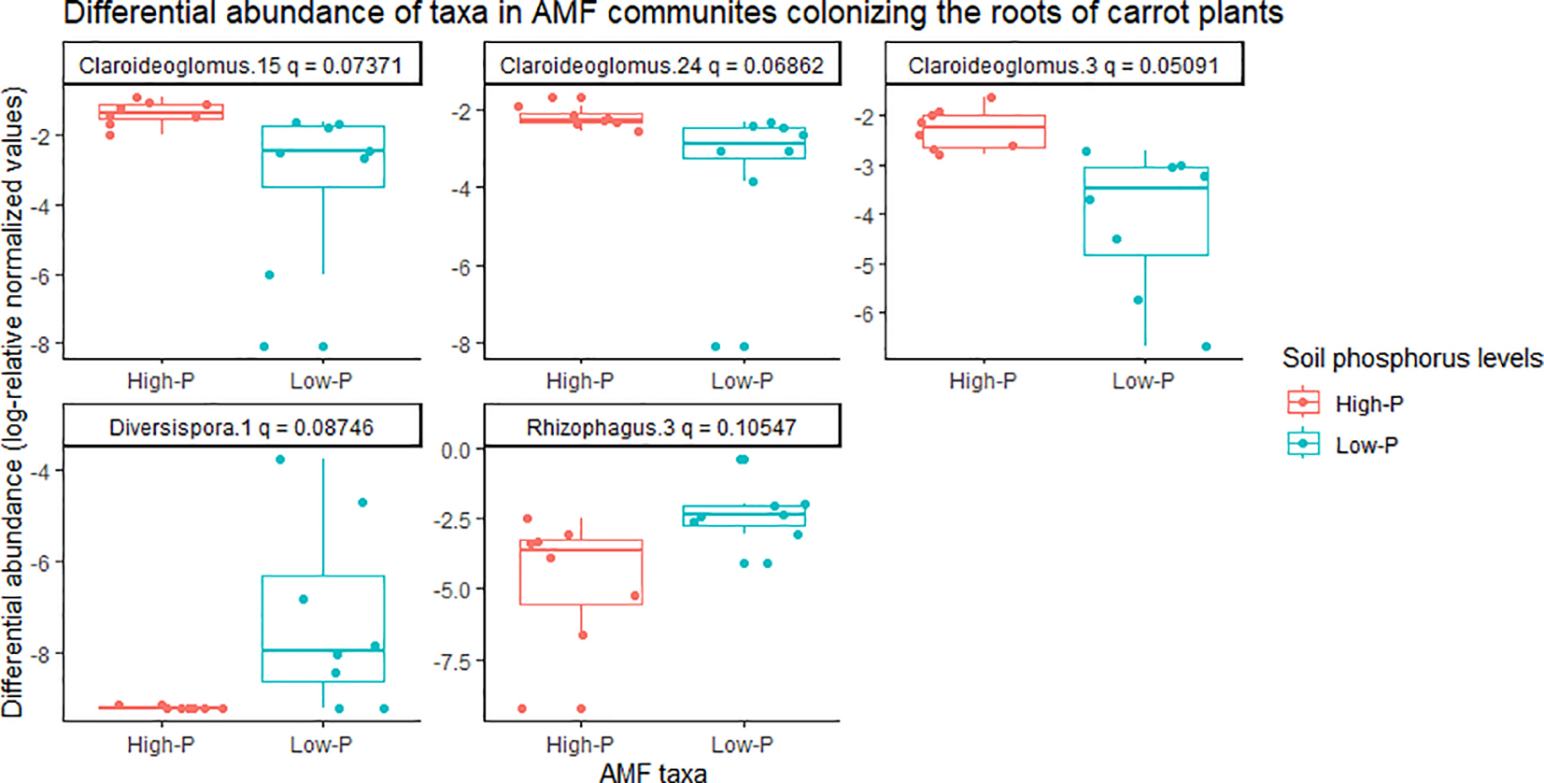
Differential abundance of arbuscular mycorrhizal fungal (AMF) communities colonizing the roots of carrot plants grown in muck soil with low- and high-phosphorus levels in a field trial in Holland Marsh, Ontario, Canada. Taxa with differences in relative abundance, based on the Wilcoxon rank-sum test (q-value ~0.1) are shown. Each taxon represents an ESV along with taxonomic assignment to the genus level. The soil pre-plant phosphorus levels were ~46 ppm in the low phosphorus treatment, and ~78 ppm in the high phosphorus treatment.

There was a significant effect of soil phosphorus levels on the composition of AMF communities colonizing carrot roots, based on PERMANOVA (*P* = 0.001) and multivariate dispersion within the groups (*P* = 0.367) (**Table 1**). The PCA plot (B) showed two distinct AMF communities, with little overlap, in the roots of carrot grown on low- vs. high-phosphorus soils (**Figure 5**). The PCA plot (B) explained a total of 72% of the variance in AMF communities, in which PC1 represented 60.4% and PC2 represented 11.7% (**Figure 5**). The relative abundance of the three taxa belonging to *Claroideoglomus* was greater in the roots of carrot grown in high-phosphorus soil than in those grown in low-phosphorus soil (**Figure 6**). The richness of AMF communities colonizing the roots of carrots grown in low-phosphorus soil was significantly greater than that of onions grown in either low- or high-phosphorus soil. There were no significant differences in the mean richness or Shannon’s and Inverse Simpson’s diversity indices of AMF communities detected in the roots of carrot plants growing on low- vs. high-phosphorus soils (**Table 2**).

## Discussion

Application of the AMF inoculant, *R. irregularis* to onion seeds increased the diversity of AMF communities colonizing the roots of onion plants. This was unexpected because *R. irregularis* was the only AMF species in the commercial inoculant. However, this treatment appeared to have influenced the composition of AMF communities colonizing the roots. The same AMF inoculant did not have any effect on the carrot seeds. Thus, applying the *R. irregularis* inoculant had a different effect on the diversity and composition of AMF communities colonizing the roots of the two plant species belonging to two very different families (Apiaceae and Amaryllidaceae).

The increase in the richness of AMF communities colonizing the roots of onion plants grown from *R. irregularis*-coated seeds was unexpected because we hypothesized that the AMF inoculant would reduce AMF diversity as a result of containing a single species of AMF. The relative abundance of *Rhizophagus* species was approximately two times greater in the roots of onion plants grown from AMF-coated seeds than in those grown from untreated seeds. *Rhizophagus* inoculant may have outcompeted native AMF in the field soil to colonize the roots of onion plants, as competition within AMF communities can exclude some AMF species (Hepper et al., 1988). Adding AMF inoculant to the seeds was expected to reduce the richness of AMF communities colonizing the roots of both onion and carrot plants. It is important to mention that this study was one year in duration. Further research is required to confirm this effect of AMF inoculant as a seed coating on the richness of AMF communities colonizing the roots of onion vs. carrot. Contrary to these findings, Mummey et al. (2009) reported reduced richness of AMF communities colonizing the roots of oxeye daisy (*Leucanthemum vulgare* L.) in a pot experiment in a growth chamber with application of an AMF inoculant consisting of *Glomus deserticola* Trappe, Bloss and Menge or *G. claroideum* Schenck and Sm. However, Antunes et al. (2009) reported no effect of a commercial AMF inoculant containing *R. irregularis* (Premier Tech., Quebec, Canada) on the composition of native AMF communities colonizing the roots of maize (*Zea mays* L.) plants in pot experiments in a greenhouse. The product they tested was produced by the same company that treated onion and carrot seeds. The findings of Antunes et al. (2009) support the results of this study, which showed no effect of AMF inoculant on the AMF communities colonizing the roots of carrot. However, the lack of an effect of commercial AMF inoculant on native AMF communities should be interpreted with caution because there may be a lag between the application of AMF inoculant and its effects on native AMF communities (Sakai et al., 2001). Based on these results, further research, especially in field experiments, is recommended to determine the effects of AMF inoculants on the diversity of native AMF communities that colonize the roots of various crops.

In this study, the soil phosphorus level influenced the AMF communities colonizing the roots of the carrots. The plots had two levels of pre-plant soil phosphorus: high (~78 ppm) and relatively low (~46 ppm). According to the OMAFRA guidelines for classifying soils based on phosphorus levels, soils with approximately 46 ppm of phosphorus are categorized as having a moderate phosphorus level (OMAFRA, 2017). Most of the growers’ fields in Ontario have a soil phosphorus level of more than 60 ppm (OMAFRA, 2017). In comparison to the soil phosphorus levels in the growers’ fields, plots with phosphorus levels of ~46 ppm in this study were considered a low-phosphorus soils. Onion and carrot were seeded in adjacent plots with each soil phosphorus level, with a 2-m-wide border separating the onion plots from the carrot plots. Soil phosphorus levels influenced the AMF communities colonizing the roots of carrots, but there was no significant effect on onion plants based on the PERMANOVA. Multivariate dispersion validated the results of the PERMANOVA. However, soil phosphorus levels had no effect on richness and diversity based on diversity indices. Therefore, it is difficult to conclude whether diversity is affected by soil phosphorus levels. Gosling et al. (2013) reported variations in response of AMF communities colonizing the roots of three plant species in relation to soil phosphorus levels in the same field. The diversity of AMF communities colonizing the roots of maize and soybean (*Glycine max* L.) decreased with an increase in soil phosphorus level, but there was no effect on field violet (*Viola arvensis* L.) grown in the same sandy loam soil in Warwick, UK, with phosphorus content ranging from 10 ppm to 280 ppm (Gosling et al., 2013). The negative effect of high soil phosphorus on AMF communities colonizing roots has been reported by many researchers, e.g., du Toit et al. (2019); Hijiri et al. (2006), and van Geel et al. (2015). Some studies have demonstrated the negative effect of soil phosphorus on AMF in roots, measured as a reduction in the percentage of root colonization by AMF using microscopic methods. du Toit et al. (2019) evaluated the effect of soil phosphorus levels on percentage root colonization of plants onion grown in a steam-pasteurized sandy soil to inactivate any native AMF and other soil microorganisms, inoculated with commercial AMF product (AGTIV Premier Tech., Quebec, Canada) in a growth chamber. Application of phosphorus (superphosphate) to increase the soil phosphorus level to 40 ppm and 80 ppm decreased the percentage of root colonization by AMF compared to no phosphorus application (~20 ppm). In this field study in Canada, the percentage of root colonization by AMF in onion and carrot plants grown in soil with low and high phosphorus levels was quantified microscopically as part of a separate project, which showed 40%–50% of root colonization of both crops, but no effect of soil phosphorus levels on the extent of root colonization of either crop (Ilyas et al., 2018).

The relative abundance of AMF genera detected in carrot roots was influenced by soil phosphorus levels. The abundance of *Claroideoglomus* was greater in AMF communities colonizing the roots of carrots grown in high- vs. low-phosphorus soils. A recent study by Chen et al. (2023) also reported the effects of soil phosphorous (adenosine monophosphate applied at 20 mg kg^−1^ soil) and host species on AMF communities detected in the roots of the perennial herbs *Solidago canadensis* L. and *S. decurrens*, compared to soil with no phosphorus applied in a greenhouse study. The application of phosphorus increased the relative abundance of Claroideoglomeraceae, and decreased Acaulosporaceae in AMF communities colonizing the roots of *S. decurrens* (Chen et al., 2023). In contrast, application of phosphorus increased the relative abundance of Glomeraceae, and decreased the abundance of Diversisporaceae and Acaulosporaceae in *S. canadensis* (Chen et al., 2023).

In this field study in Ontario, Canada, plant species was the most influential factor affecting AMF communities colonizing the roots of onion and carrot in high organic matter (muck) soil. These results strongly support the hypothesis that onion and carrot plants grown in the same ecological zone, even in the same field, can develop different AMF communities in their roots. Onions and carrots are two vegetables extensively grown in the Holland Marsh. Onions are monocotyledonous species belonging to the family Amaryllidaceae, and carrots are eudicotyledonous species belonging to the family Apiaceae (Angiosperm Phylogeny Group III, 2009). Overall, the AMF communities of carrots were richer and more diverse than those of onions. The effects of plant species on AMF communities colonizing the roots have been reported by other researchers. Bainard et al. (2014) reported that the diversity of AMF communities colonizing roots differed in wheat, pea, and lentil plants growing in a field on silt-loam soil in Saskatchewan, Canada. Geoffroy et al. (2017) reported that the richness and composition of AMF colonizing the roots of taro (*Colocasia esculenta* L.) plants were different from those of *Pterocarpus* species in a swamp forest in Guadeloupe. Vandenkoornhuyse et al. (2003) reported that the diversity of AMF communities differed among grass species coexisting in the same plot in a semi-natural hill pasture near Kelso, UK. Klironomos (2002) hypothesized that host plants can influence the composition and diversity of AMF communities in roots by selecting AMF species that benefit plants in particular environments. Broeckling et al. (2007) related changes in microbial communities in the rhizosphere to the root exudates of the host. Similarly, Higo et al. (2015) revealed that different patterns of root exudation from host plants influenced the diversity of AMF communities. A study of root exudates of onion and carrot, and how these exudates influence AMF communities in roots, would be a promising future research. Future studies are required to understand how AMF inoculants compete and interact with native AMF colonizing the roots of various plant species. AMF produce lipochitoligosaccharides, known as “Myc factors,” that are involved in establishment of AMF symbiosis with plant roots (Weidmann et al., 2004; Kuhn et al., 2010). It is not known how Myc factors might affect competition between native and non-native AMF isolates.

In these fungi colonizing the roots of onion and carrot plants were identified at the genus level by Illumina sequencing. The cutoff for genus identification was based on a confidence threshold value of ≥90 for ESVs. Several AMF were identified as species, but most ESVs had confidence threshold values of <90. A cut-off for species identification with a confidence threshold of ≥90 would have removed most of the data from the analysis. Consequently, all data were included in the analysis, but identification was limited to the genus level, and identified species were termed as “potential species.” The DNA fragment amplified by the primer pair AMV 4.5NF-AMDGR was approximately 280 bp, making it well suited for Illumina sequencing. Shorter sequences cannot provide accurate taxonomic resolution for species-level identification (Hoggard et al., 2018; Lucking et al., 2020).

In this study, the AMF colonizing the roots of onion and carrot plants belonged to three orders and three families. Five genera were identified in the roots of both onion and carrot: *Diversispora*, *Claroideoglomus*, *Funneliformis*, *Rhizophagus*, and *Glomus. G. macrocarpum* Tul. and C. Tul. was identified only in the roots of the onions. These results are comparable to those of Galván et al. (2009) and Knerr et al. (2018), who idenfied AMF genera that colonize the roots of onion crops under field conditions. Galván et al. (2009) reported *Glomus*, *Rhizophagus*, *Archaeospora*, and *Paraglomus* in AMF communities colonizing the roots of onion plants grown on clay soils in the Netherlands, which were identified using the NS5–ITS4i primer pair with RFLP analysis. Knerr et al. (2018) reported *Glomus*, *Rhizophagus*, *Claroideoglomus*, *Acaulospora*, *Diversispora*, and *Paraglomus* in the AMF communities colonizing the roots of onion crops on very sandy soils in the Columbia Basin of the Pacific Northwest USA, which were identified using the AMV 4.5NF-AMDGR primer pair by pyrosequencing. Several AMF genera were common among these studies, but *Funneliformis* was also found in this study on a muck soil in Ontario, Canada, whereas *Acaulospora* and *Paraglomus* were found in the sandy soils of the Columbia Basin, USA. *Funneliformis* has been reported in AMF communities colonize the roots of peas, lentils, and wheat in fields in Saskatchewan, Canada, using the AML1–AML2 primer pair with pyrosequencing (Bainard et al., 2014).

In the present study, *G. macrocarpum* in the AMF communities was not detected in carrot roots. Approximately 15 ESVs belonging to *G. macrocarpum* have been identified in the AMF community colonizing the roots of onion plants. *G. macrocarpum* was identified in onion root samples collected from plots of all treatments from each replicate block in the field trial, which confirmed the presence of *G. macrocarpum* throughout the field. Declerck et al. (1998) reported *in vitro* colonization of carrot roots by *G. macrocarpum*. However, the extent of colonization was limited, and only a few spores of *G. macrocarpum* were found in the AMF community colonizing the roots of carrot compared to other *Glomus* species (Declerck et al., 1998). It is possible that *G. macrocarpum* did not compete well with other AMF species colonizing the roots of carrot plants under the field conditions in this study on muck soil. Further research is needed to confirm the ability of *G. macrocarpum* to colonize the roots of carrots grown in various soil types and under different field conditions.

## Conclusion

This study provides a snapshot of the richness, diversity, and composition of AMF colonizing the roots of onion and carrot plants grown in high organic matter (muck) soil at two levels of soil phosphorus, and the application of the AMF inoculant *R. irregularis* as a seed coating. Overall, the results suggest that plant species (onion vs. carrot) were the most influential factors affecting the richness and composition of AMF communities under the conditions of this study. AMF communities colonizing the roots of carrot plants were more diverse than those of onion plants. Application of *R. irregularis* as an AMF inoculant to onion and carrot seeds increased the richness of AMF communities in onion roots, but not carrot roots, even though *R. irregularis* was found to colonize the roots of both plant species. AMF communities colonizing the roots of onion and carrot plants included *Diversispora*, *Claroideoglomus*, *Funneliformis*, *Rhizophagus*, and *Glomus* spp. The composition of AMF communities in carrot roots differed between low- and high-phosphorus soils. AMF communities had a greater abundance of *Claroideoglomus* in carrot roots growing in high-phosphorus-muck soil. Soil phosphorus levels did not affect AMF colonization of the onion roots. This study also demonstrated that naturally occurring AMF were present in both onion and carrot plants, even in the presence of relatively high soil phosphorus in agricultural soil.

## Data availability statement

The corresponding authors of this article will provide the raw data supporting their conclusions upon request, without any unnecessary restrictions or reservations. The raw sequences were submitted to NCBI database as Sequence Read Archive (SRA) and available under BioProject accession number PRJNA1051590.

## Author contributions

UI: Conceptualization, Investigation, Methodology, Software, Visualization, Writing – original draft, Writing – review & editing. LD: Writing – review & editing, Conceptualization, Methodology, Supervision, Validation, Visualization. MH: Methodology, Writing – original draft, Data curation, Software. MM: Conceptualization, Funding acquisition, Methodology, Project administration, Resources, Supervision, Validation, Visualization, Writing – original draft, Writing – review & editing.

## Supplementary material

Supplementary Figure 1 Position of binding of the primer pair AMV4.5F – AMDGR (Sato et al., 2005) on the small subunit of the 18S ribosomal RNA gene. The lines represent the region of binding by the specific primers. Arrows on the lines indicate the direction of sequencing of the primer. The dotted vertical lines represent the position of the AMV 4.5NF – AMDGR primer pair binding sites.

Supplementary Figure 2 Relative abundance of arbuscular mycorrhizal fungal (AMF) genera colonizing the roots of onion and carrot plants grown in a muck soil in a field trial in Holland Marsh, Ontario, Canada.

## References

[B1] Adholeya A. Tiwari P. Singh R. (2005). Large-scale inoculum production of arbuscular mycorrhizal fungi on root organs and inoculation strategies in In vitro culture of mycorrhizas. Eds. Declerck S. Fortin J. A. Strullu D. G. (Springer, Berlin Press, Heidelberg), 315–338. doi: 10.1007/3-540-27331-X_17

[B2] Angiosperm Phylogeny Group III (2009). An update of the Angiosperm Phylogeny Group classification for the orders and families of flowering plants: APG III. Bot. J. Linn. Soc 161, 105–121. doi: 10.1016/j.jep.2015.05.035

[B3] Antunes P. M. Koch A. M. Dunfield K. E. Hart M. M. Downing A. Rillig M. C.. (2009). Influence of commercial inoculation with *Glomus intraradices* on the structure and functioning of an AM fungal community from an agricultural site. Plant Soil 317, 257–266. doi: 10.1007/s11104-008-9806-y

[B4] Augé R. M. (2004). Arbuscular mycorrhizae and soil and plant water relations. Can. J. Soil Sci. 84, 373–381. doi: 10.4141/S04-002

[B5] Averill C. Turner B. L. Finzi A. C. (2014). Mycorrhiza-mediated competition between plants and decomposers drives soil carbon storage. Nature 505, 543–545. doi: 10.1038/nature12901 24402225

[B6] Bainard L. D. Bainard J. D. Hamel C. Gan Y. (2014). Spatial and temporal structuring of arbuscular mycorrhizal communities is differentially influenced by abiotic factors and host crop in a semi-arid prairie agroecosystem. FEMS Microbiol. Ecol. 88, 333–344. doi: 10.1111/1574-6941.12300 24527842

[B7] Barr J. (2010). Restoration of plant communities in The Netherlands through the application of arbuscular mycorrhizal fungi. Symbiosis 52, 87–94. doi: 10.1007/s13199-010-0105-z

[B8] Berruti A. Borriello R. Della Beffa M. T. Scariot V. Bianciotto V. (2013). Application of nonspecific commercial AMF inocula results in poor mycorrhization in *Camellia japonica* L. Symbiosis 61, 63–76. doi: 10.1007/s13199-013-0258-7

[B9] Błaszkowski J. García M. S. Niezgoda P. Zubek S. Fernández F. Vila A.. (2022). A new order, Entrophosporales, and three new *Entrophospora* species in Glomeromycota. Front. Microbiol. 13. doi: 10.3389/fmicb.2022.962856 PMC9835108 36643412

[B10] Broeckling C. D. Bergelson J. Vivanco J. M. Manter D. K. Broz A. K. (2007). Root exudates regulate soil fungal community composition and diversity. Appl. Environ. Microbiol. 74, 738–744. doi: 10.1128/aem.02188-07 18083870 PMC2227741

[B11] Campos-Soriano L. García-Martínez J. Segundo B. S. (2012). The arbuscular mycorrhizal symbiosis promotes the systemic induction of regulatory defence-related genes in rice leaves and confers resistance to pathogen infection. Mol. Plant Pathol. 13, 579–592. doi: 10.1111/j.1364-3703.2011.00773.x 22212404 PMC6638712

[B12] Chandrasekaran M. Chanratana M. Kim K. Seshadri S. Sa T. (2019). Impact of arbuscular mycorrhizal fungi on photosynthesis, water status, and gas exchange of plants under salt stress–a meta-analysis. Front. Plant Sci. 10. doi: 10.3389/fpls.2019.00457 PMC6476944 31040857

[B13] Chen L. Wang M. Shi Y. Ma P. Xiao Y. Yu H.. (2023). Soil phosphorus form affects the advantages that arbuscular mycorrhizal fungi confer on the invasive plant species, *Solidago canadensis*, over its congener. Front. Microbiol. 14. doi: 10.3389/fmicb.2023.1160631 PMC10140316 37125154

[B14] Chen S. Zhao H. Zou C. Li Y. Chen Y. Wang Z.. (2017). Combined inoculation with multiple arbuscular mycorrhizal fungi improves growth, nutrient uptake and photosynthesis in cucumber seedlings. Front. Microbiol. 8. doi: 10.3389/fmicb.2017.02516 PMC5742139 29312217

[B15] Declerck S. Strullu D. G. Plenchette C. (1998). Monoxenic culture of the intraradical forms of *Glomus* species isolated from a tropical ecosystem: a proposed methodology for germplasm collection. Mycologia 90, 579. doi: 10.2307/3761216

[B16] Dickie I. A. Martínez-García L. B. Koele N. Grelet G. A. Tylianakis J. M. Peltzer D. A.. (2013). Mycorrhizas and mycorrhizal fungal communities throughout ecosystem development. Plant Soil 367, 11–39. doi: 10.1007/s11104-013-1609-0

[B17] du Toit L. J. Derie M. L. Holmes B. J. Morgan P. Brouwer L. R. Waters T. D. (2019). The influence of soil phosphorus levels on onion root colonization by mycorrhizal fungi from commercial inoculants 2017. Plant Dis. Manage. Rep. 13, V009.

[B18] Edgar R. C. (2016). UNOISE2: improved error-correction for Illumina 16S and ITS amplicon sequencing. bioRxiv 15, 81257. doi: 10.1101/081257

[B19] Egan C. P. (2017). Community structure of arbuscular mycorrhizal fungi along an elevation gradient. [PhD thesis (Canad (BC: University of British Columbia).

[B20] Ehsanfar S. Modarres-Sanavy S. A. (2005). Crop protection by seed coating. Appl. Biol. Sci. 70, 225–229. 16637182

[B21] Fiorilli V. Catoni M. Miozzi L. Novero M. Accotto G. P. Lanfranco L. (2009). Global and cell-type gene expression profiles in tomato plants colonized by an arbuscular mycorrhizal fungus. New Phytol. 184, 975–987. doi: 10.1111/j.1469-8137.2009.03031.x 19765230

[B22] Fritz M. Jakobsen I. Lyngkjær M. F. Thordal-Christensen H. Pons-Kühnemann J. (2006). Arbuscular mycorrhiza reduces susceptibility of tomato to *Alternaria solani* . Mycorrhiza 16, 413–419. doi: 10.1007/s00572-006-0051-z 16614816

[B23] Galván G. A. Parádi I. Burger K. Baar J. Kuyper T. W. Scholten O. E.. (2009). Molecular diversity of arbuscular mycorrhizal fungi in onion roots from organic and conventional farming systems in the Netherlands. Mycorrhiza 19, 317–328. doi: 10.1007/s00572-009-0237-2 19301039 PMC2687515

[B24] Garmendia I. Mangas V. J. (2014). Comparative study of substrate-based and commercial formulations of arbuscular mycorrhizal fungi in romaine lettuce subjected to salt stress. J. Plant Nutr. 37, 1717–1731. doi: 10.1080/01904167.2014.889149

[B25] Geoffroy A. Sanguin H. Galiana A. Bâ A. (2017). Molecular characterization of arbuscular mycorrhizal fungi in an agroforestry system reveals the predominance of *Funneliformis* species associated with *Colocasia esculenta* and *Pterocarpus officinalis* adult trees and seedlings. Front. Microbiol. 8. doi: 10.3389/fmicb.2017.01426 PMC5532380 28804479

[B26] Gollotte A. van Tuinen D. Atkinson D. (2004). Diversity of arbuscular mycorrhizal fungi colonising roots of the grass species *Agrostis capillaris* and *Lolium perenne* in a field experiment. Mycorrhiza 14, 111–117. doi: 10.1007/s00572-003-0244-7 12768382

[B27] Gosling P. Hodge A. Goodlass G. Bending G. D. (2006). Arbuscular mycorrhizal fungi and organic farming. Agric. Ecosyst. Environ. 113, 17–35. doi: 10.1016/j.agee.2005.09.009

[B28] Gosling P. Mead A. Proctor M. Hammond J. P. Bending G. D. (2013). Contrasting arbuscular mycorrhizal communities colonizing different host plants show a similar response to a soil phosphorus concentration gradient. New Phytol. 198, 546–556. doi: 10.1111/nph.12169 23421495 PMC3798118

[B29] Hepper C. M. Azcon-aguilar C. Rosendahl S. Sen R. (1988). Competition between three species of *Glomus* used as spatially separated introduced and indigenous mycorrhizal inocula for leek (*Allium porrum* L). New Phytol. 110, 207–215. doi: 10.1111/j.1469-8137.1988.tb00254.x

[B30] Higo M. Ichida M. Isobe K. Torigoe Y. Matsuda Y. (2015). Influence of sowing season and host crop identity on the community structure of arbuscular mycorrhizal fungi colonizing roots of two different gramineous and leguminous crop species. Adv. Microbiol. 05, 107–116. doi: 10.4236/aim.2015.52011

[B31] Hijiri I. Sykorova Z. Oehl F. Ineichen K. Mader P. Wiemken A.. (2006). Communities of arbuscular mycorrhizal fungi in arable soils are not necessarily low in diversity. Mol. Ecol. 15, 2277–2289. doi: 10.1111/j.1365-294X.2006.02921.x 16780440

[B32] Hoggard M. Vesty A. Wong G. Montgomery J. M. Fourie C. Douglas R. G.. (2018). Characterizing the human mycobiota: A comparison of small subunit rRNA, ITS1, ITS2, and large subunit rRNA genomic targets. Front. Microbiol. 9. doi: 10.3389/fmicb.2018.02208 PMC6157398 30283425

[B33] Humphreys C. P. Franks P. J. Rees M. Bidartondo M. I. Leake J. R. Beerling D. J. (2010). Mutualistic mycorrhiza-like symbiosis in the most ancient group of land plants. Nat. Commun. 1, 103. doi: 10.1038/ncomms1105 21045821

[B34] Ilyas U. MN. R. du Toit L. J. McDonald M. (2018). Effect of mycorrhizae on phosphorus requirments of carrot grown on muck soil 2017. Muck vegetable cultivar trial & Research report (University of Guelph, Guelph, Canada). 61–66.

[B35] Johnson N. C. (2010). Resource stoichiometry elucidates the structure and function of arbuscular mycorrhizas across scales. New Phytol. 185, 631–647. doi: 10.1111/j.1469-8137.2009.03110.x 19968797

[B36] Kennedy P. G. Mielke L. A. Nguyen N. H. (2018). Ecological responses to forest age, habitat, and host vary by mycorrhizal type in boreal peatlands. Mycorrhiza 28, 315–328. doi: 10.1007/s00572-018-0821-4 29504037

[B37] Klironomos J. N. (2002). Feedback with soil biota contributes to plant rarity and invasiveness in communities. Nature 417, 67–70. doi: 10.1038/417067a 11986666

[B38] Knerr A. J. Wheeler D. Schlatter D. Sharma-Poudyal D. du Toit L. J. Paulitz T. C. (2018). Arbuscular mycorrhizal fungal communities in organic and conventional onion crops in the Columbia Basin of the Pacific Northwest United States. Phytobiomes J. 2, 194–207. doi: 10.1094/pbiomes-05-18-0022-r

[B39] Koorem K. Uibopuu A. Saks Ü. Zobel M. Öpik M. Gazol A.. (2014). Soil nutrient content influences the abundance of soil microbes but not plant biomass at the small-scale. PloS One 9, e91998. doi: 10.1371/journal.pone.0091998 24637633 PMC3956881

[B40] Kuhn H. Küster H. Requena N. (2010). Membrane steroid-binding protein 1 induced by a diffusible fungal signal is critical for mycorrhization in *Medicago truncatula* . New Phytol. 185, 716–733. doi: 10.1111/j.1469-8137.2009.03116.x 20003073

[B41] Liu W. Zhang Y. Jiang S. Deng Y. Christie P. Murray P. J.. (2016). Arbuscular mycorrhizal fungi in soil and roots respond differently to phosphorus inputs in an intensively managed calcareous agricultural soil. Sci. Rep. 6, 24902. doi: 10.1038/srep24902 27102357 PMC4840358

[B42] Lucking R. L. Aime M. C. Robbertse B. Miller A. N. Ariyawansa H. A. Aoki T.. (2020). Unambiguous identification of fungi: Where do we stand and how accurate and precise is fungal DNA barcoding? IMA Fungus 11, 14. doi: 10.14806/ej.17.1.200 32714773 PMC7353689

[B43] Mckinney M. L. Lockwood J. L. (1999). Biotic homogenization, a few winners replacing many losers in the next mass extinction. Trends Ecol. Evol. 14, 450–453. doi: 10.1016/S0169-5347(99)01679-1 10511724

[B44] Montesinos E. Moragrega C. Llorente I. Vilardell P. (1995). Susceptibility of selected pear cultivars to infection by *Stemphylium vesicarium* and the influence of leaf and fruit age. Plant Dis. 79, 471–473. doi: 10.1094/PD-79-0471

[B45] Morton J. B. Benny G. L. (1990). Revised classification of arbuscular mycorrhizal fungi (Zygomycetes): a new order, Glomales, two new suborders, Glomineae and Gigasporineae, and two new families, Acaulosporaceae and Gigasporaceae, with an emendation of Glomaceae. Mycotaxon 37, 471–491.

[B46] Morton J. B. Redecker D. (2001). Two new families of Glomales, Archaeosporaceae and Paraglomaceae, with two new genera *Archaeospora* and *Paraglomus*, based on concordant molecular and morphological characters. Mycologia 93, 181–195. doi: 10.2307/3761615

[B47] Mummey D. L. Antunes P. M. Rillig M. C. (2009). Arbuscular mycorrhizal fungi pre-inoculant identity determines community composition in roots. Soil Biol. Biochem. 41, 1173–1179. doi: 10.1016/j.soilbio.2009.02.027

[B48] O’Callaghan M. (2016). Microbial inoculation of seed for improved crop performance: issues and opportunities. Appl. Microbiol. Biotechnol. 100, 5729–5746. doi: 10.1007/s00253-016-7590-9 27188775 PMC4909795

[B49] Oehl F. Sieverding E. Palenzuela J. Ineichen K. da silva G. (2011). Advances in Glomeromycota taxonomy and classificatio. IMA Fungus 2, 191–199. doi: 10.5598/imafungus.2011.02.02.10 22679604 PMC3359817

[B50] OMAFRA. (2017). Publication 838, vegetable crop protection guide (Ontario Ministry of Agriculture, Food and Rural Affairs). Available at: http://www.omafra.gov.on.ca/english/crops/pub838/pub838.pdf (Accessed 2019 Feb. 20).

[B51] Palenzuela J. Ferrol N. Boller T. Azcón-Aguilar C. Oehl F. (2008). *Otospora bareai*, a new fungal species in the Glomeromycetes from a dolomitic shrub land in Sierra de Baza National Park (Granada, Spain). Mycologia 100, 296–305. doi: 10.3852/mycologia.100.2.296 18592903

[B52] Pozo M. J. Azcón-Aguilar C. (2007). Unraveling mycorrhiza-induced resistance. Curr. Opin. Plant Biol. 10, 393–398. doi: 10.1016/j.pbi.2007.05.004 17658291

[B53] Qi S. Wang J. Wan L. Dai Z. da Matos D. M. Du D.. (2022). Arbuscular mycorrhizal fungi contribute to phosphorous uptake and allocation strategies of *Solidago canadensis* in a phosphorous-deficient environment. Front. Plant Sci. 13. doi: 10.3389/fpls.2022.831654 PMC8987128 35401639

[B54] Redecker D. Hijri I. Wiemken A. (2003). Molecular identification of arbuscular mycorrhizae perspective and problems. Folia Geobot. 38, 113–124. doi: 10.1007/BF02803144

[B55] Rocha I. Ma Y. Souza-Alonso P. Vosátka M. Freitas H. Oliveira R. S. (2019). seed coating: a tool for delivering beneficial microbes to agricultural crops. Front. Plant Sci. 10. doi: 10.3389/fpls.2019.01357 PMC6852281 31781135

[B56] Rognes T. Flouri T. Nichols B. Quince C. Mahé F. (2016). VSEARCH: a versatile open source tool for metagenomics. PeerJ 4, e2584. doi: 10.7717/peerj.2584 27781170 PMC5075697

[B57] Sakai A. K. Allendorf F. W. Holt J. S. Lodge M. Molofsky J. With K. A.. (2001). The population biology of invasive species. Annu. Rev. Ecol. Syst. 32, 305–332. doi: 10.1146/annurev.ecolsys.32.081501.114037

[B58] Sato K. Suyama Y. Saito M. Sugawara K. (2005). A new primer for discrimination of arbuscular mycorrhizal fungi with polymerase chain reaction-denature gradient gel electrophoresis. Grassl. Sci. 51, 179–181. doi: 10.1111/j.1744-697X.2005.00023.x

[B59] Schenck N. C. Smith G. S. (1982). Additional new and unreported species of mycorrhizal fungi (Endogonaceae) from Florida. Mycologia 74, 77–92. doi: 10.1080/00275514.1982.12021472

[B60] Schüßler A. Walker C. (2010). The Glomeromycota: A species list with new families and new genera. Schüßler, A., Walker, C., .pg 59. Available at :http://www.amf-phylogeny.com.

[B61] Schüßler A. Walker C. (2010). The Glomeromycota: A species list with new families and new genera. Schüßler A. Walker C. Gloucester, published in libraries at Royal Botanic Garden Edinburgh, Kew, Botanische Staatssammlung Munich, and Oregon State University 59. Available at: http://www.amf-phylogeny.com.

[B62] Schußler D. Walker C. Schu A. (2001). A new fungal phylum, the Glomeromycota. Mycol. Res. 105, 1413–1421. doi: 10.1017/S0953756201005196

[B63] Schwartz M. W. Hoeksema J. D. Gehring C. A. Johnson N. C. Klironomos J. N. Abbott L. K.. (2006). The promise and the potential consequences of the global transport of mycorrhizal fungal inoculum. Ecol. Lett. 9, 501–515. doi: 10.1111/j.1461-0248.2006.00910.x 16643296

[B64] Siddiqui Z. A. Akhtar M. S. (2007). Biocontrol of a chickpea root rot disease complex with phosphate-solubilizing microorganisms. J. Plant Pathol. 89, 67–77. doi: 10.4454/jpp.v89i1.725

[B65] Sieverding E. Oehl F. (2006). Revision of *Entrophospora* and description of *Kuklospora* and *Intraspora*, two new genera in the arbuscular mycorrhizal Glomeromycetes. J. Appl. Bot. Food Qual. 80, 69–81.

[B66] Smith S. E. Read D. J. (2008). Mycorrhizal symbiosis. 3rd edition (SAn Diego, CA: Academic Press).

[B67] Spatafora J. W. Chang Y. Benny G. L. Lazarus K. Smith M. E. Berbee M. L.. (2016). A phylum-level phylogenetic classification of zygomycete fungi based on genome-scale data. Mycologia 108, 1028–1046. doi: 10.3852/16-042 27738200 PMC6078412

[B68] Stockinger H. Walker C. Schüßler A. (2009). *Glomus intraradices* DAOM197198”, a model fungus in arbuscular mycorrhiza research, is not *Glomus intraradices* . New Phytol. 183, 1176–1187. doi: 10.1111/j.1469-8137.2009.02874.x 19496945

[B69] Sun Z. Song J. Xin X. Xie X. Zhao B. (2018). Arbuscular mycorrhizal fungal 14-3-3 proteins are involved in arbuscule formation and responses to abiotic stresses during AM symbiosis. Front. Microbiol. 9. doi: 10.3389/fmicb.2018.00091 PMC5844941 29556216

[B70] Vályi K. Mardhiah U. Rillig M. C. Hempel S. (2016). Community assembly and coexistence in communities of arbuscular mycorrhizal fungi. ISME J. 10, 2341–2351. doi: 10.1038/ismej.2016.46 27093046 PMC5030697

[B71] Vandenkoornhuyse P. Ridgway K. P. Watson I. J. Fitter A. H. Young J. P. W. (2003). Co-existing grass species have distinctive arbuscular mycorrhizal communities. Mol. Ecol. 12, 3085–3095. doi: 10.1046/j.1365-294X.2003.01967.x 14629388

[B72] van Geel M. Ceustermans A. Van Hemelrijck W. Lievens B. Honnay O. (2015). Decrease in diversity and changes in community composition of arbuscular mycorrhizal fungi in roots of apple trees with increasing orchard management intensity across a regional scale. Mol. Ecol. 24, 941–952. doi: 10.1111/mec.13079 25586038

[B73] Walder F. van der Heijden M. G. A. (2015). Regulation of resource exchange in the arbuscular mycorrhizal symbiosis. Nat. Plants 1, 1515. doi: 10.1038/nplants.2015.159 27251530

[B74] Walker C. Schüßler A. Vincent B. Cranenbrouck S. Declerck S. (2021). Anchoring the species *Rhizophagus intraradices* (formerly *Glomus intraradices*). Fungal Syst. Evol. 8, 179–201. doi: 10.3114/fuse.2021.08.14 35005581 PMC8687058

[B75] Walker C. Vestberg M. Schüßler A. (2007). Nomenclatural clarifications in glomeromycota. Mycol. Res. 111, 253–255. doi: 10.1016/j.mycres.2007.02.009 17324754

[B76] Wang Q. Garrity G. M. Tiedje J. M. Cole J. R. (2007). Naive Bayesian Classifier for rapid assignment of rRNA sequences into the new bacterial taxonomy. Appl. Environ. Microbiol. 73, 5261–5267. doi: 10.1128/AEM.00062-07 17586664 PMC1950982

[B77] Weidmann S. Sanchez L. Descombin J. Chatagnier O. Gianinazzi S. Gianinazzi-Pearson V. (2004). Fungal elicitation of signal transduction-related plant genes precedes mycorrhiza establishment and requires the *dmi3* gene in *Medicago truncatula* . Mol. Plant-Microbe Interact. 17, 1385–1393. doi: 10.1094/MPMI.2004.17.12.1385 15597744

[B78] Weiss S. Xu Z. Z. Peddada S. Amir A. Bittinger K. Gonzalez A.. (2017). Normalization and microbial differential abundance strategies depend upon data characteristics. Microbiome 5, 5–27. doi: 10.1186/s40168-017-0237-y 28253908 PMC5335496

[B79] Wu S. C. Cao Z. H. Li Z. G. Cheung K. C. Wong M. H. (2005). Effects of biofertilizer containing N-fixer, P and K solubilizers and AM fungi on maize growth: A greenhouse trial. Geoderma 125, 155–166. doi: 10.1016/j.geoderma.2004.07.003

