# Supplementary material for: Influence of plant species, mycorrhizal inoculant, and soil phosphorus level on arbuscular mycorrhizal communities in onion and carrot roots
Source: Front Plant Sci. 2024 Jan 15;14:1324626. doi: 10.3389/fpls.2023.1324626 (PMC10823018; doi:10.3389/fpls.2023.1324626)
Supplement: Supplementary file 1 [file Table_1.pdf]

Supplementary Table S1. Taxonomy assignment of exact sequence variant (ESV) of arbuscular mycorrhizal fungi at genus-level with a confidence threshold values ≥ 90 identified using primer pair AMV4.5NF – AMDGR in the roots of onions and carrot plants grown on muck soil in a field trial at Holland Marsh, Ontario

ESV	Crop	Reads	Class	Order	Family	Genus	BP	Species	BP
DG_Otu1	Carrot	995	Glomeromycetes	Glomerales	Glomeraceae	*Funneliformis*	1	*Funneliformis_mosseae*	1
DG_Otu10	Carrot	28	Glomeromycetes	Diversisporales	Diversisporaceae	*Diversispora*	1	*Diversispora_sp_W2423*	1
DG_Otu111	Carrot	38	Glomeromycetes	Glomerales	Glomerales_uncultured	*Glomerales_undefined*	0.95	*Glomeromycotina_sp_8536*	0.92
DG_Otu12	Carrot	8	Glomeromycetes	Diversisporales	Diversisporaceae	*Diversispora*	1	*Diversispora_celata*	0.99
DG_Otu13	Carrot	1187	Glomeromycetes	Claroideoglomusles	Claroideoglomeraceae	*Claroideoglomus*	1	*Glomeromycotina_sp_MIB_8381*	0.93
DG_Otu14	Carrot	361	Glomeromycetes	Glomerales	Glomeraceae	*Rhizophagus*	1	*Rhizophagus_irregularis*	0.55
DG_Otu148	Carrot	7	Glomeromycetes	Glomerales	Glomerales_uncultured	*Glomerales_undefined*	0.91	*Glomeromycotina_sp_8536*	0.88
DG_Otu15	Carrot	732	Glomeromycetes	Glomerales	Glomeraceae	*Rhizophagus*	1	*Rhizophagus_irregularis*	0.97
DG_Otu164	Carrot	3	Glomeromycetes	Claroideoglomusles	Claroideoglomeraceae	*Claroideoglomus*	0.97	*Glomus_sp_NBR_PP1*	0.85
DG_Otu18	Carrot	377	Glomeromycetes	Claroideoglomusles	Claroideoglomeraceae	*Claroideoglomus*	1	*Glomeromycotina_sp_MIB_8381*	1
DG_Otu19	Carrot	923	Glomeromycetes	Claroideoglomusles	Claroideoglomeraceae	*Claroideoglomus*	0.97	*Glomus_sp_NBR_PP1*	0.95
DG_Otu2	Carrot	5944	Glomeromycetes	Glomerales	Glomeraceae	*Rhizophagus*	1	*Glomus_sp_MUCL_43194*	0.55
DG_Otu20	Carrot	372	Glomeromycetes	Claroideoglomusles	Claroideoglomeraceae	*Claroideoglomus*	0.9	*Glomus_sp_NBR_PP1*	0.85
DG_Otu3	Carrot	2194	Glomeromycetes	Glomerales	Glomeraceae	*Rhizophagus*	1	*Rhizophagus_irregularis*	0.96
DG_Otu30	Carrot	119	Glomeromycetes	Claroideoglomusles	Claroideoglomeraceae	*Claroideoglomus*	1	*Claroideoglomus_etunicatum*	0.32
DG_Otu37	Carrot	327	Glomeromycetes	Claroideoglomusles	Claroideoglomeraceae	*Claroideoglomus*	0.94	*Glomus_sp_NBR_PP1*	0.92
DG_Otu4	Carrot	2604	Glomeromycetes	Glomerales	Glomeraceae	*Rhizophagus*	1	*Rhizophagus_irregularis*	0.86
DG_Otu5	Carrot	2209	Glomeromycetes	Claroideoglomusles	Claroideoglomeraceae	*Claroideoglomus*	1	*Glomeromycotina_sp_MIB_8381*	0.58
DG_Otu6	Carrot	1779	Glomeromycetes	Claroideoglomusles	Claroideoglomeraceae	*Claroideoglomus*	0.94	*Claroideoglomus_etunicatum*	0.66
DG_Otu65	Carrot	147	Glomeromycetes	Glomerales	Glomerales_uncultured	*Glomerales_undefined*	0.96	*Glomeromycotina_sp_8536*	0.85
DG_Otu7	Carrot	1664	Glomeromycetes	Glomerales	Glomeraceae	*Rhizophagus*	1	*Rhizophagus_irregularis*	0.64
DG_Otu76	Carrot	110	Glomeromycetes	Claroideoglomusles	Claroideoglomeraceae	*Claroideoglomus*	0.91	*Glomus_sp_NBR_PP1*	0.87
DG_Otu85	Carrot	74	Glomeromycetes	Glomerales	Glomerales_uncultured	*Glomerales_undefined*	0.92	*Glomeromycotina_sp_8536*	0.89
DG_Otu9	Carrot	2419	Glomeromycetes	Claroideoglomusles	Claroideoglomeraceae	*Claroideoglomus*	0.99	*Glomeromycotina_sp_MIB_8381*	0.55
DG_Otu10	Carrot	26	Glomeromycetes	Diversisporales	Diversisporaceae	*Diversispora*	1	*Diversispora_sp_W2423*	1
DG_Otu13	Carrot	871	Glomeromycetes	Claroideoglomusles	Claroideoglomeraceae	*Claroideoglomus*	1	*Glomeromycotina_sp_MIB_8381*	0.93
DG_Otu14	Carrot	1277	Glomeromycetes	Glomerales	Glomeraceae	*Rhizophagus*	1	*Rhizophagus_irregularis*	0.55
DG_Otu15	Carrot	277	Glomeromycetes	Glomerales	Glomeraceae	*Rhizophagus*	1	*Rhizophagus_irregularis*	0.97
DG_Otu16	Carrot	6	Glomeromycetes	Diversisporales	Diversisporaceae	*Diversispora*	1	*Diversispora_celata*	0.97
DG_Otu19	Carrot	7	Glomeromycetes	Claroideoglomusles	Claroideoglomeraceae	*Claroideoglomus*	0.97	*Glomus_sp_NBR_PP1*	0.95
DG_Otu2	Carrot	179	Glomeromycetes	Glomerales	Glomeraceae	*Rhizophagus*	1	*Glomus_sp_MUCL_43194*	0.55
DG_Otu20	Carrot	425	Glomeromycetes	Claroideoglomusles	Claroideoglomeraceae	*Claroideoglomus*	0.9	*Glomus_sp_NBR_PP1*	0.85
DG_Otu3	Carrot	332	Glomeromycetes	Glomerales	Glomeraceae	*Rhizophagus*	1	*Rhizophagus_irregularis*	0.96
DG_Otu4	Carrot	697	Glomeromycetes	Glomerales	Glomeraceae	*Rhizophagus*	1	*Rhizophagus_irregularis*	0.86
DG_Otu5	Carrot	1261	Glomeromycetes	Claroideoglomusles	Claroideoglomeraceae	*Claroideoglomus*	1	*Glomeromycotina_sp_MIB_8381*	0.58
DG_Otu6	Carrot	2664	Glomeromycetes	Claroideoglomusles	Claroideoglomeraceae	*Claroideoglomus*	0.94	*Claroideoglomus_etunicatum*	0.66

DG_Otu7	Carrot	346	Glomeromycetes	Glomerales	Glomeraceae	*Rhizophagus*	1	*Rhizophagus_irregularis*	0.64
DG_Otu9	Carrot	992	Glomeromycetes	Claroideoglomusles	Claroideoglomeraceae	*Claroideoglomus*	0.99	*Glomeromycotina_sp_MIB_8381*	0.55
DG_Otu1	Carrot	7	Glomeromycetes	Glomerales	Glomeraceae	*Funneliformis*	1	*Funneliformis_mosseae*	1
DG_Otu122	Carrot	29	Glomeromycetes	Claroideoglomusles	Claroideoglomeraceae	*Claroideoglomus*	0.9	*Glomus_sp_NBR_PP1*	0.9
DG_Otu13	Carrot	14	Glomeromycetes	Claroideoglomusles	Claroideoglomeraceae	*Claroideoglomus*	1	*Glomeromycotina_sp_MIB_8381*	0.93
DG_Otu14	Carrot	164	Glomeromycetes	Glomerales	Glomeraceae	*Rhizophagus*	1	*Rhizophagus_irregularis*	0.55
DG_Otu2	Carrot	126	Glomeromycetes	Glomerales	Glomeraceae	*Rhizophagus*	1	*Glomus_sp_MUCL_43194*	0.55
DG_Otu27	Carrot	725	Glomeromycetes	Claroideoglomusles	Claroideoglomeraceae	*Claroideoglomus*	0.92	*Glomus_sp_NBR_PP1*	0.92
DG_Otu3	Carrot	3111	Glomeromycetes	Glomerales	Glomeraceae	*Rhizophagus*	1	*Rhizophagus_irregularis*	0.96
DG_Otu4	Carrot	5	Glomeromycetes	Glomerales	Glomeraceae	*Rhizophagus*	1	*Rhizophagus_irregularis*	0.86
DG_Otu5	Carrot	10	Glomeromycetes	Claroideoglomusles	Claroideoglomeraceae	*Claroideoglomus*	1	*Glomeromycotina_sp_MIB_8381*	0.58
DG_Otu6	Carrot	7	Glomeromycetes	Claroideoglomusles	Claroideoglomeraceae	*Claroideoglomus*	0.94	*Claroideoglomus_etunicatum*	0.66
DG_Otu7	Carrot	149	Glomeromycetes	Glomerales	Glomeraceae	*Rhizophagus*	1	*Rhizophagus_irregularis*	0.64
DG_Otu9	Carrot	216	Glomeromycetes	Claroideoglomusles	Claroideoglomeraceae	*Claroideoglomus*	0.99	*Glomeromycotina_sp_MIB_8381*	0.55
DG_Otu13	Carrot	2124	Glomeromycetes	Claroideoglomusles	Claroideoglomeraceae	*Claroideoglomus*	1	*Glomeromycotina_sp_MIB_8381*	0.93
DG_Otu14	Carrot	202	Glomeromycetes	Glomerales	Glomeraceae	*Rhizophagus*	1	*Rhizophagus_irregularis*	0.55
DG_Otu15	Carrot	9	Glomeromycetes	Glomerales	Glomeraceae	*Rhizophagus*	1	*Rhizophagus_irregularis*	0.97
DG_Otu19	Carrot	53	Glomeromycetes	Claroideoglomusles	Claroideoglomeraceae	*Claroideoglomus*	0.97	*Glomus_sp_NBR_PP1*	0.95
DG_Otu2	Carrot	424	Glomeromycetes	Glomerales	Glomeraceae	*Rhizophagus*	1	*Glomus_sp_MUCL_43194*	0.55
DG_Otu20	Carrot	281	Glomeromycetes	Claroideoglomusles	Claroideoglomeraceae	*Claroideoglomus*	0.9	*Glomus_sp_NBR_PP1*	0.85
DG_Otu21	Carrot	45	Glomeromycetes	Claroideoglomusles	Claroideoglomeraceae	*Claroideoglomus*	0.93	*Glomus_sp_NBR_PP1*	0.91
DG_Otu26	Carrot	33	Glomeromycetes	Claroideoglomusles	Claroideoglomeraceae	*Claroideoglomus*	0.95	*Glomus_sp_NBR_PP1*	0.93
DG_Otu3	Carrot	13	Glomeromycetes	Glomerales	Glomeraceae	*Rhizophagus*	1	*Rhizophagus_irregularis*	0.96
DG_Otu4	Carrot	216	Glomeromycetes	Glomerales	Glomeraceae	*Rhizophagus*	1	*Rhizophagus_irregularis*	0.86
DG_Otu5	Carrot	2402	Glomeromycetes	Claroideoglomusles	Claroideoglomeraceae	*Claroideoglomus*	1	*Glomeromycotina_sp_MIB_8381*	0.58
DG_Otu6	Carrot	2684	Glomeromycetes	Claroideoglomusles	Claroideoglomeraceae	*Claroideoglomus*	0.94	*Claroideoglomus_etunicatum*	0.66
DG_Otu7	Carrot	188	Glomeromycetes	Glomerales	Glomeraceae	*Rhizophagus*	1	*Rhizophagus_irregularis*	0.64
DG_Otu9	Carrot	2026	Glomeromycetes	Claroideoglomusles	Claroideoglomeraceae	*Claroideoglomus*	0.99	*Glomeromycotina_sp_MIB_8381*	0.55
DG_Otu1	Carrot	662	Glomeromycetes	Glomerales	Glomeraceae	*Funneliformis*	1	*Funneliformis_mosseae*	1
DG_Otu10	Carrot	61	Glomeromycetes	Diversisporales	Diversisporaceae	*Diversispora*	1	*Diversispora_sp_W2423*	1
DG_Otu13	Carrot	1642	Glomeromycetes	Claroideoglomusles	Claroideoglomeraceae	*Claroideoglomus*	1	*Glomeromycotina_sp_MIB_8381*	0.93
DG_Otu14	Carrot	1591	Glomeromycetes	Glomerales	Glomeraceae	*Rhizophagus*	1	*Rhizophagus_irregularis*	0.55
DG_Otu15	Carrot	1240	Glomeromycetes	Glomerales	Glomeraceae	*Rhizophagus*	1	*Rhizophagus_irregularis*	0.97
DG_Otu16	Carrot	10	Glomeromycetes	Diversisporales	Diversisporaceae	*Diversispora*	1	*Diversispora_celata*	0.97
DG_Otu19	Carrot	237	Glomeromycetes	Claroideoglomusles	Claroideoglomeraceae	*Claroideoglomus*	0.97	*Glomus_sp_NBR_PP1*	0.95
DG_Otu2	Carrot	3643	Glomeromycetes	Glomerales	Glomeraceae	*Rhizophagus*	1	*Glomus_sp_MUCL_43194*	0.55
DG_Otu20	Carrot	80	Glomeromycetes	Claroideoglomusles	Claroideoglomeraceae	*Claroideoglomus*	0.9	*Glomus_sp_NBR_PP1*	0.85
DG_Otu21	Carrot	293	Glomeromycetes	Claroideoglomusles	Claroideoglomeraceae	*Claroideoglomus*	0.93	*Glomus_sp_NBR_PP1*	0.91
DG_Otu26	Carrot	79	Glomeromycetes	Claroideoglomusles	Claroideoglomeraceae	*Claroideoglomus*	0.95	*Glomus_sp_NBR_PP1*	0.93
DG_Otu28	Carrot	202	Glomeromycetes	Claroideoglomusles	Claroideoglomeraceae	*Claroideoglomus*	0.98	*Glomus_sp_NBR_PP1*	0.97
DG_Otu3	Carrot	1529	Glomeromycetes	Glomerales	Glomeraceae	*Rhizophagus*	1	*Rhizophagus_irregularis*	0.96

DG_Otu4	Carrot	5033	Glomeromycetes	Glomerales	Glomeraceae	*Rhizophagus*	1	*Rhizophagus_irregularis*	0.86
DG_Otu43	Carrot	162	Glomeromycetes	Glomerales	Glomerales_uncultured	*Glomerales_undefined*	0.92	*Glomeromycotina_sp_8536*	0.81
DG_Otu46	Carrot	41	Glomeromycetes	Claroideoglomusles	Claroideoglomeraceae	*Claroideoglomus*	0.95	*Glomus_sp_NBR_PP1*	0.89
DG_Otu47	Carrot	224	Glomeromycetes	Glomerales	Glomerales_uncultured	*Glomerales_undefined*	0.97	*Glomeromycotina_sp_8536*	0.96
DG_Otu5	Carrot	5652	Glomeromycetes	Claroideoglomusles	Claroideoglomeraceae	*Claroideoglomus*	1	*Glomeromycotina_sp_MIB_8381*	0.58
DG_Otu6	Carrot	4988	Glomeromycetes	Claroideoglomusles	Claroideoglomeraceae	*Claroideoglomus*	0.94	*Claroideoglomus_etunicatum*	0.66
DG_Otu7	Carrot	3284	Glomeromycetes	Glomerales	Glomeraceae	*Rhizophagus*	1	*Rhizophagus_irregularis*	0.64
DG_Otu9	Carrot	2343	Glomeromycetes	Claroideoglomusles	Claroideoglomeraceae	*Claroideoglomus*	0.99	*Glomeromycotina_sp_MIB_8381*	0.55
DG_Otu92	Carrot	60	Glomeromycetes	Claroideoglomusles	Claroideoglomeraceae	*Claroideoglomus*	0.98	*Glomus_sp_NBR_PP1*	0.97
DG_Otu13	Carrot	5142	Glomeromycetes	Claroideoglomusles	Claroideoglomeraceae	*Claroideoglomus*	1	*Glomeromycotina_sp_MIB_8381*	0.93
DG_Otu14	Carrot	5	Glomeromycetes	Glomerales	Glomeraceae	*Rhizophagus*	1	*Rhizophagus_irregularis*	0.55
DG_Otu15	Carrot	6	Glomeromycetes	Glomerales	Glomeraceae	*Rhizophagus*	1	*Rhizophagus_irregularis*	0.97
DG_Otu16	Carrot	100	Glomeromycetes	Diversisporales	Diversisporaceae	*Diversispora*	1	*Diversispora_celata*	0.97
DG_Otu18	Carrot	844	Glomeromycetes	Claroideoglomusles	Claroideoglomeraceae	*Claroideoglomus*	1	*Glomeromycotina_sp_MIB_8381*	1
DG_Otu19	Carrot	40	Glomeromycetes	Claroideoglomusles	Claroideoglomeraceae	*Claroideoglomus*	0.97	*Glomus_sp_NBR_PP1*	0.95
DG_Otu2	Carrot	83	Glomeromycetes	Glomerales	Glomeraceae	*Rhizophagus*	1	*Glomus_sp_MUCL_43194*	0.55
DG_Otu30	Carrot	115	Glomeromycetes	Claroideoglomusles	Claroideoglomeraceae	*Claroideoglomus*	1	*Claroideoglomus_etunicatum*	0.32
DG_Otu4	Carrot	2141	Glomeromycetes	Glomerales	Glomeraceae	*Rhizophagus*	1	*Rhizophagus_irregularis*	0.86
DG_Otu5	Carrot	####	Glomeromycetes	Claroideoglomusles	Claroideoglomeraceae	*Claroideoglomus*	1	*Glomeromycotina_sp_MIB_8381*	0.58
DG_Otu6	Carrot	6171	Glomeromycetes	Claroideoglomusles	Claroideoglomeraceae	*Claroideoglomus*	0.94	*Claroideoglomus_etunicatum*	0.66
DG_Otu7	Carrot	509	Glomeromycetes	Glomerales	Glomeraceae	*Rhizophagus*	1	*Rhizophagus_irregularis*	0.64
DG_Otu9	Carrot	4997	Glomeromycetes	Claroideoglomusles	Claroideoglomeraceae	*Claroideoglomus*	0.99	*Glomeromycotina_sp_MIB_8381*	0.55
DG_Otu1	Carrot	10	Glomeromycetes	Glomerales	Glomeraceae	*Funneliformis*	1	*Funneliformis_mosseae*	1
DG_Otu10	Carrot	37	Glomeromycetes	Diversisporales	Diversisporaceae	*Diversispora*	1	*Diversispora_sp_W2423*	1
DG_Otu11	Carrot	3	Glomeromycetes	Glomerales	Glomeraceae	*Funneliformis*	1	*Funneliformis_mosseae*	0.99
DG_Otu13	Carrot	3	Glomeromycetes	Claroideoglomusles	Claroideoglomeraceae	*Claroideoglomus*	1	*Glomeromycotina_sp_MIB_8381*	0.93
DG_Otu14	Carrot	6	Glomeromycetes	Glomerales	Glomeraceae	*Rhizophagus*	1	*Rhizophagus_irregularis*	0.55
DG_Otu2	Carrot	1656	Glomeromycetes	Glomerales	Glomeraceae	*Rhizophagus*	1	*Glomus_sp_MUCL_43194*	0.55
DG_Otu3	Carrot	444	Glomeromycetes	Glomerales	Glomeraceae	*Rhizophagus*	1	*Rhizophagus_irregularis*	0.96
DG_Otu4	Carrot	10	Glomeromycetes	Glomerales	Glomeraceae	*Rhizophagus*	1	*Rhizophagus_irregularis*	0.86
DG_Otu6	Carrot	316	Glomeromycetes	Claroideoglomusles	Claroideoglomeraceae	*Claroideoglomus*	0.94	*Claroideoglomus_etunicatum*	0.66
DG_Otu7	Carrot	648	Glomeromycetes	Glomerales	Glomeraceae	*Rhizophagus*	1	*Rhizophagus_irregularis*	0.64
DG_Otu1	Carrot	181	Glomeromycetes	Glomerales	Glomeraceae	*Funneliformis*	1	*Funneliformis_mosseae*	1
DG_Otu10	Carrot	201	Glomeromycetes	Diversisporales	Diversisporaceae	*Diversispora*	1	*Diversispora_sp_W2423*	1
DG_Otu13	Carrot	1752	Glomeromycetes	Claroideoglomusles	Claroideoglomeraceae	*Claroideoglomus*	1	*Glomeromycotina_sp_MIB_8381*	0.93
DG_Otu14	Carrot	5	Glomeromycetes	Glomerales	Glomeraceae	*Rhizophagus*	1	*Rhizophagus_irregularis*	0.55
DG_Otu15	Carrot	306	Glomeromycetes	Glomerales	Glomeraceae	*Rhizophagus*	1	*Rhizophagus_irregularis*	0.97
DG_Otu18	Carrot	363	Glomeromycetes	Claroideoglomusles	Claroideoglomeraceae	*Claroideoglomus*	1	*Glomeromycotina_sp_MIB_8381*	1
DG_Otu19	Carrot	224	Glomeromycetes	Claroideoglomusles	Claroideoglomeraceae	*Claroideoglomus*	0.97	*Glomus_sp_NBR_PP1*	0.95
DG_Otu2	Carrot	859	Glomeromycetes	Glomerales	Glomeraceae	*Rhizophagus*	1	*Glomus_sp_MUCL_43194*	0.55
DG_Otu21	Carrot	414	Glomeromycetes	Claroideoglomusles	Claroideoglomeraceae	*Claroideoglomus*	0.93	*Glomus_sp_NBR_PP1*	0.91

DG_Otu26	Carrot	420	Glomeromycetes	Claroideoglomusles	Claroideoglomeraceae	*Claroideoglomus*	0.95	*Glomus_sp_NBR_PP1*	0.93
DG_Otu3	Carrot	877	Glomeromycetes	Glomerales	Glomeraceae	*Rhizophagus*	1	*Rhizophagus_irregularis*	0.96
DG_Otu30	Carrot	43	Glomeromycetes	Claroideoglomusles	Claroideoglomeraceae	*Claroideoglomus*	1	*Claroideoglomus_etunicatum*	0.32
DG_Otu4	Carrot	1640	Glomeromycetes	Glomerales	Glomeraceae	*Rhizophagus*	1	*Rhizophagus_irregularis*	0.86
DG_Otu46	Carrot	120	Glomeromycetes	Claroideoglomusles	Claroideoglomeraceae	*Claroideoglomus*	0.95	*Glomus_sp_NBR_PP1*	0.89
DG_Otu5	Carrot	7831	Glomeromycetes	Claroideoglomusles	Claroideoglomeraceae	*Claroideoglomus*	1	*Glomeromycotina_sp_MIB_8381*	0.58
DG_Otu6	Carrot	6295	Glomeromycetes	Claroideoglomusles	Claroideoglomeraceae	*Claroideoglomus*	0.94	*Claroideoglomus_etunicatum*	0.66
DG_Otu7	Carrot	595	Glomeromycetes	Glomerales	Glomeraceae	*Rhizophagus*	1	*Rhizophagus_irregularis*	0.64
DG_Otu9	Carrot	2312	Glomeromycetes	Claroideoglomusles	Claroideoglomeraceae	*Claroideoglomus*	0.99	*Glomeromycotina_sp_MIB_8381*	0.55
DG_Otu93	Carrot	59	Glomeromycetes	Claroideoglomusles	Claroideoglomeraceae	*Claroideoglomus*	0.94	*Glomus_sp_NBR_PP1*	0.86
DG_Otu1	Carrot	253	Glomeromycetes	Glomerales	Glomeraceae	*Funneliformis*	1	*Funneliformis_mosseae*	1
DG_Otu13	Carrot	950	Glomeromycetes	Claroideoglomusles	Claroideoglomeraceae	*Claroideoglomus*	1	*Glomeromycotina_sp_MIB_8381*	0.93
DG_Otu14	Carrot	1226	Glomeromycetes	Glomerales	Glomeraceae	*Rhizophagus*	1	*Rhizophagus_irregularis*	0.55
DG_Otu15	Carrot	820	Glomeromycetes	Glomerales	Glomeraceae	*Rhizophagus*	1	*Rhizophagus_irregularis*	0.97
DG_Otu19	Carrot	173	Glomeromycetes	Claroideoglomusles	Claroideoglomeraceae	*Claroideoglomus*	0.97	*Glomus_sp_NBR_PP1*	0.95
DG_Otu2	Carrot	6242	Glomeromycetes	Glomerales	Glomeraceae	*Rhizophagus*	1	*Glomus_sp_MUCL_43194*	0.55
DG_Otu20	Carrot	837	Glomeromycetes	Claroideoglomusles	Claroideoglomeraceae	*Claroideoglomus*	0.9	*Glomus_sp_NBR_PP1*	0.85
DG_Otu3	Carrot	2977	Glomeromycetes	Glomerales	Glomeraceae	*Rhizophagus*	1	*Rhizophagus_irregularis*	0.96
DG_Otu4	Carrot	1743	Glomeromycetes	Glomerales	Glomeraceae	*Rhizophagus*	1	*Rhizophagus_irregularis*	0.86
DG_Otu5	Carrot	4254	Glomeromycetes	Claroideoglomusles	Claroideoglomeraceae	*Claroideoglomus*	1	*Glomeromycotina_sp_MIB_8381*	0.58
DG_Otu6	Carrot	1569	Glomeromycetes	Claroideoglomusles	Claroideoglomeraceae	*Claroideoglomus*	0.94	*Claroideoglomus_etunicatum*	0.66
DG_Otu7	Carrot	225	Glomeromycetes	Glomerales	Glomeraceae	*Rhizophagus*	1	*Rhizophagus_irregularis*	0.64
DG_Otu9	Carrot	2094	Glomeromycetes	Claroideoglomusles	Claroideoglomeraceae	*Claroideoglomus*	0.99	*Glomeromycotina_sp_MIB_8381*	0.55
DG_Otu1	Carrot	59	Glomeromycetes	Glomerales	Glomeraceae	*Funneliformis*	1	*Funneliformis_mosseae*	1
DG_Otu13	Carrot	1591	Glomeromycetes	Claroideoglomusles	Claroideoglomeraceae	*Claroideoglomus*	1	*Glomeromycotina_sp_MIB_8381*	0.93
DG_Otu14	Carrot	1065	Glomeromycetes	Glomerales	Glomeraceae	*Rhizophagus*	1	*Rhizophagus_irregularis*	0.55
DG_Otu15	Carrot	538	Glomeromycetes	Glomerales	Glomeraceae	*Rhizophagus*	1	*Rhizophagus_irregularis*	0.97
DG_Otu19	Carrot	65	Glomeromycetes	Claroideoglomusles	Claroideoglomeraceae	*Claroideoglomus*	0.97	*Glomus_sp_NBR_PP1*	0.95
DG_Otu2	Carrot	3096	Glomeromycetes	Glomerales	Glomeraceae	*Rhizophagus*	1	*Glomus_sp_MUCL_43194*	0.55
DG_Otu20	Carrot	400	Glomeromycetes	Claroideoglomusles	Claroideoglomeraceae	*Claroideoglomus*	0.9	*Glomus_sp_NBR_PP1*	0.85
DG_Otu28	Carrot	61	Glomeromycetes	Claroideoglomusles	Claroideoglomeraceae	*Claroideoglomus*	0.98	*Glomus_sp_NBR_PP1*	0.97
DG_Otu3	Carrot	420	Glomeromycetes	Glomerales	Glomeraceae	*Rhizophagus*	1	*Rhizophagus_irregularis*	0.96
DG_Otu30	Carrot	59	Glomeromycetes	Claroideoglomusles	Claroideoglomeraceae	*Claroideoglomus*	1	*Claroideoglomus_etunicatum*	0.32
DG_Otu4	Carrot	1187	Glomeromycetes	Glomerales	Glomeraceae	*Rhizophagus*	1	*Rhizophagus_irregularis*	0.86
DG_Otu5	Carrot	6178	Glomeromycetes	Claroideoglomusles	Claroideoglomeraceae	*Claroideoglomus*	1	*Glomeromycotina_sp_MIB_8381*	0.58
DG_Otu6	Carrot	2667	Glomeromycetes	Claroideoglomusles	Claroideoglomeraceae	*Claroideoglomus*	0.94	*Claroideoglomus_etunicatum*	0.66
DG_Otu64	Carrot	159	Glomeromycetes	Claroideoglomusles	Claroideoglomeraceae	*Claroideoglomus*	0.95	*Glomus_sp_NBR_PP1*	0.93
DG_Otu7	Carrot	944	Glomeromycetes	Glomerales	Glomeraceae	*Rhizophagus*	1	*Rhizophagus_irregularis*	0.64
DG_Otu70	Carrot	121	Glomeromycetes	Claroideoglomusles	Claroideoglomeraceae	*Claroideoglomus*	0.99	*Glomus_sp_NBR_PP1*	0.99
DG_Otu89	Carrot	70	Glomeromycetes	Claroideoglomusles	Claroideoglomeraceae	*Claroideoglomus*	0.95	*Glomus_sp_NBR_PP1*	0.94
DG_Otu9	Carrot	2293	Glomeromycetes	Claroideoglomusles	Claroideoglomeraceae	*Claroideoglomus*	0.99	*Glomeromycotina_sp_MIB_8381*	0.55

DG_Otu1	Carrot	93	Glomeromycetes	Glomerales	Glomeraceae	*Funneliformis*	1	*Funneliformis_mosseae*	1
DG_Otu109	Carrot	39	Glomeromycetes	Claroideoglomusles	Claroideoglomeraceae	*Claroideoglomus*	0.99	*Glomus_sp_NBR_PP1*	0.99
DG_Otu12	Carrot	22	Glomeromycetes	Diversisporales	Diversisporaceae	*Diversispora*	1	*Diversispora_celata*	0.99
DG_Otu13	Carrot	2527	Glomeromycetes	Claroideoglomusles	Claroideoglomeraceae	*Claroideoglomus*	1	*Glomeromycotina_sp_MIB_8381*	0.93
DG_Otu14	Carrot	2264	Glomeromycetes	Glomerales	Glomeraceae	*Rhizophagus*	1	*Rhizophagus_irregularis*	0.55
DG_Otu15	Carrot	1654	Glomeromycetes	Glomerales	Glomeraceae	*Rhizophagus*	1	*Rhizophagus_irregularis*	0.97
DG_Otu17	Carrot	14	Glomeromycetes	Diversisporales	Diversisporaceae	*Diversispora*	1	*Diversispora_sp_W2423*	0.9
DG_Otu19	Carrot	238	Glomeromycetes	Claroideoglomusles	Claroideoglomeraceae	*Claroideoglomus*	0.97	*Glomus_sp_NBR_PP1*	0.95
DG_Otu2	Carrot	5878	Glomeromycetes	Glomerales	Glomeraceae	*Rhizophagus*	1	*Glomus_sp_MUCL_43194*	0.55
DG_Otu20	Carrot	120	Glomeromycetes	Claroideoglomusles	Claroideoglomeraceae	*Claroideoglomus*	0.9	*Glomus_sp_NBR_PP1*	0.85
DG_Otu21	Carrot	136	Glomeromycetes	Claroideoglomusles	Claroideoglomeraceae	*Claroideoglomus*	0.93	*Glomus_sp_NBR_PP1*	0.91
DG_Otu28	Carrot	229	Glomeromycetes	Claroideoglomusles	Claroideoglomeraceae	*Claroideoglomus*	0.98	*Glomus_sp_NBR_PP1*	0.97
DG_Otu3	Carrot	2666	Glomeromycetes	Glomerales	Glomeraceae	*Rhizophagus*	1	*Rhizophagus_irregularis*	0.96
DG_Otu4	Carrot	3931	Glomeromycetes	Glomerales	Glomeraceae	*Rhizophagus*	1	*Rhizophagus_irregularis*	0.86
DG_Otu43	Carrot	17	Glomeromycetes	Glomerales	Glomerales_uncultured	*Glomerales_undefined*	0.92	*Glomeromycotina_sp_8536*	0.81
DG_Otu5	Carrot	7182	Glomeromycetes	Claroideoglomusles	Claroideoglomeraceae	*Claroideoglomus*	1	*Glomeromycotina_sp_MIB_8381*	0.58
DG_Otu58	Carrot	54	Glomeromycetes	Claroideoglomusles	Claroideoglomeraceae	*Claroideoglomus*	0.99	*Glomus_sp_NBR_PP1*	0.99
DG_Otu6	Carrot	3166	Glomeromycetes	Claroideoglomusles	Claroideoglomeraceae	*Claroideoglomus*	0.94	*Claroideoglomus_etunicatum*	0.66
DG_Otu7	Carrot	2733	Glomeromycetes	Glomerales	Glomeraceae	*Rhizophagus*	1	*Rhizophagus_irregularis*	0.64
DG_Otu78	Carrot	57	Glomeromycetes	Claroideoglomusles	Claroideoglomeraceae	*Claroideoglomus*	0.99	*Glomus_sp_NBR_PP1*	0.99
DG_Otu9	Carrot	3113	Glomeromycetes	Claroideoglomusles	Claroideoglomeraceae	*Claroideoglomus*	0.99	*Glomeromycotina_sp_MIB_8381*	0.55
DG_Otu1	Carrot	305	Glomeromycetes	Glomerales	Glomeraceae	*Funneliformis*	1	*Funneliformis_mosseae*	1
DG_Otu13	Carrot	3039	Glomeromycetes	Claroideoglomusles	Claroideoglomeraceae	*Claroideoglomus*	1	*Glomeromycotina_sp_MIB_8381*	0.93
DG_Otu14	Carrot	229	Glomeromycetes	Glomerales	Glomeraceae	*Rhizophagus*	1	*Rhizophagus_irregularis*	0.55
DG_Otu15	Carrot	661	Glomeromycetes	Glomerales	Glomeraceae	*Rhizophagus*	1	*Rhizophagus_irregularis*	0.97
DG_Otu18	Carrot	355	Glomeromycetes	Claroideoglomusles	Claroideoglomeraceae	*Claroideoglomus*	1	*Glomeromycotina_sp_MIB_8381*	1
DG_Otu19	Carrot	532	Glomeromycetes	Claroideoglomusles	Claroideoglomeraceae	*Claroideoglomus*	0.97	*Glomus_sp_NBR_PP1*	0.95
DG_Otu2	Carrot	4045	Glomeromycetes	Glomerales	Glomeraceae	*Rhizophagus*	1	*Glomus_sp_MUCL_43194*	0.55
DG_Otu20	Carrot	310	Glomeromycetes	Claroideoglomusles	Claroideoglomeraceae	*Claroideoglomus*	0.9	*Glomus_sp_NBR_PP1*	0.85
DG_Otu21	Carrot	260	Glomeromycetes	Claroideoglomusles	Claroideoglomeraceae	*Claroideoglomus*	0.93	*Glomus_sp_NBR_PP1*	0.91
DG_Otu3	Carrot	1323	Glomeromycetes	Glomerales	Glomeraceae	*Rhizophagus*	1	*Rhizophagus_irregularis*	0.96
DG_Otu4	Carrot	1242	Glomeromycetes	Glomerales	Glomeraceae	*Rhizophagus*	1	*Rhizophagus_irregularis*	0.86
DG_Otu47	Carrot	14	Glomeromycetes	Glomerales	Glomerales_uncultured	*Glomerales_undefined*	0.97	*Glomeromycotina_sp_8536*	0.96
DG_Otu5	Carrot	6065	Glomeromycetes	Claroideoglomusles	Claroideoglomeraceae	*Claroideoglomus*	1	*Glomeromycotina_sp_MIB_8381*	0.58
DG_Otu6	Carrot	4432	Glomeromycetes	Claroideoglomusles	Claroideoglomeraceae	*Claroideoglomus*	0.94	*Claroideoglomus_etunicatum*	0.66
DG_Otu7	Carrot	1358	Glomeromycetes	Glomerales	Glomeraceae	*Rhizophagus*	1	*Rhizophagus_irregularis*	0.64
DG_Otu9	Carrot	2753	Glomeromycetes	Claroideoglomusles	Claroideoglomeraceae	*Claroideoglomus*	0.99	*Glomeromycotina_sp_MIB_8381*	0.55
DG_Otu1	Carrot	223	Glomeromycetes	Glomerales	Glomeraceae	*Funneliformis*	1	*Funneliformis_mosseae*	1
DG_Otu10	Carrot	749	Glomeromycetes	Diversisporales	Diversisporaceae	*Diversispora*	1	*Diversispora_sp_W2423*	1
DG_Otu12	Carrot	242	Glomeromycetes	Diversisporales	Diversisporaceae	*Diversispora*	1	*Diversispora_celata*	0.99
DG_Otu13	Carrot	291	Glomeromycetes	Claroideoglomusles	Claroideoglomeraceae	*Claroideoglomus*	1	*Glomeromycotina_sp_MIB_8381*	0.93

DG_Otu14	Carrot	980	Glomeromycetes	Glomerales	Glomeraceae	*Rhizophagus*	1	*Rhizophagus_irregularis*	0.55
DG_Otu15	Carrot	696	Glomeromycetes	Glomerales	Glomeraceae	*Rhizophagus*	1	*Rhizophagus_irregularis*	0.97
DG_Otu16	Carrot	24	Glomeromycetes	Diversisporales	Diversisporaceae	*Diversispora*	1	*Diversispora_celata*	0.97
DG_Otu17	Carrot	13	Glomeromycetes	Diversisporales	Diversisporaceae	*Diversispora*	1	*Diversispora_sp_W2423*	0.9
DG_Otu18	Carrot	531	Glomeromycetes	Claroideoglomusles	Claroideoglomeraceae	*Claroideoglomus*	1	*Glomeromycotina_sp_MIB_8381*	1
DG_Otu19	Carrot	353	Glomeromycetes	Claroideoglomusles	Claroideoglomeraceae	*Claroideoglomus*	0.97	*Glomus_sp_NBR_PP1*	0.95
DG_Otu2	Carrot	159	Glomeromycetes	Glomerales	Glomeraceae	*Rhizophagus*	1	*Glomus_sp_MUCL_43194*	0.55
DG_Otu21	Carrot	993	Glomeromycetes	Claroideoglomusles	Claroideoglomeraceae	*Claroideoglomus*	0.93	*Glomus_sp_NBR_PP1*	0.91
DG_Otu26	Carrot	235	Glomeromycetes	Claroideoglomusles	Claroideoglomeraceae	*Claroideoglomus*	0.95	*Glomus_sp_NBR_PP1*	0.93
DG_Otu3	Carrot	404	Glomeromycetes	Glomerales	Glomeraceae	*Rhizophagus*	1	*Rhizophagus_irregularis*	0.96
DG_Otu4	Carrot	9969	Glomeromycetes	Glomerales	Glomeraceae	*Rhizophagus*	1	*Rhizophagus_irregularis*	0.86
DG_Otu5	Carrot	1765	Glomeromycetes	Claroideoglomusles	Claroideoglomeraceae	*Claroideoglomus*	1	*Glomeromycotina_sp_MIB_8381*	0.58
DG_Otu6	Carrot	3006	Glomeromycetes	Claroideoglomusles	Claroideoglomeraceae	*Claroideoglomus*	0.94	*Claroideoglomus_etunicatum*	0.66
DG_Otu67	Carrot	134	Glomeromycetes	Claroideoglomusles	Claroideoglomeraceae	*Claroideoglomus*	0.95	*Glomus_sp_NBR_PP1*	0.95
DG_Otu7	Carrot	4620	Glomeromycetes	Glomerales	Glomeraceae	*Rhizophagus*	1	*Rhizophagus_irregularis*	0.64
DG_Otu9	Carrot	546	Glomeromycetes	Claroideoglomusles	Claroideoglomeraceae	*Claroideoglomus*	0.99	*Glomeromycotina_sp_MIB_8381*	0.55
DG_Otu13	Carrot	1814	Glomeromycetes	Claroideoglomusles	Claroideoglomeraceae	*Claroideoglomus*	1	*Glomeromycotina_sp_MIB_8381*	0.93
DG_Otu14	Carrot	803	Glomeromycetes	Glomerales	Glomeraceae	*Rhizophagus*	1	*Rhizophagus_irregularis*	0.55
DG_Otu15	Carrot	310	Glomeromycetes	Glomerales	Glomeraceae	*Rhizophagus*	1	*Rhizophagus_irregularis*	0.97
DG_Otu19	Carrot	66	Glomeromycetes	Claroideoglomusles	Claroideoglomeraceae	*Claroideoglomus*	0.97	*Glomus_sp_NBR_PP1*	0.95
DG_Otu2	Carrot	9765	Glomeromycetes	Glomerales	Glomeraceae	*Rhizophagus*	1	*Glomus_sp_MUCL_43194*	0.55
DG_Otu20	Carrot	175	Glomeromycetes	Claroideoglomusles	Claroideoglomeraceae	*Claroideoglomus*	0.9	*Glomus_sp_NBR_PP1*	0.85
DG_Otu21	Carrot	138	Glomeromycetes	Claroideoglomusles	Claroideoglomeraceae	*Claroideoglomus*	0.93	*Glomus_sp_NBR_PP1*	0.91
DG_Otu3	Carrot	2506	Glomeromycetes	Glomerales	Glomeraceae	*Rhizophagus*	1	*Rhizophagus_irregularis*	0.96
DG_Otu4	Carrot	2085	Glomeromycetes	Glomerales	Glomeraceae	*Rhizophagus*	1	*Rhizophagus_irregularis*	0.86
DG_Otu44	Carrot	179	Glomeromycetes	Claroideoglomusles	Claroideoglomeraceae	*Claroideoglomus*	0.99	*Glomus_sp_NBR_PP1*	0.96
DG_Otu5	Carrot	5420	Glomeromycetes	Claroideoglomusles	Claroideoglomeraceae	*Claroideoglomus*	1	*Glomeromycotina_sp_MIB_8381*	0.58
DG_Otu58	Carrot	126	Glomeromycetes	Claroideoglomusles	Claroideoglomeraceae	*Claroideoglomus*	0.99	*Glomus_sp_NBR_PP1*	0.99
DG_Otu6	Carrot	1633	Glomeromycetes	Claroideoglomusles	Claroideoglomeraceae	*Claroideoglomus*	0.94	*Claroideoglomus_etunicatum*	0.66
DG_Otu7	Carrot	1835	Glomeromycetes	Glomerales	Glomeraceae	*Rhizophagus*	1	*Rhizophagus_irregularis*	0.64
DG_Otu9	Carrot	2196	Glomeromycetes	Claroideoglomusles	Claroideoglomeraceae	*Claroideoglomus*	0.99	*Glomeromycotina_sp_MIB_8381*	0.55
DG_Otu1	Carrot	1231	Glomeromycetes	Glomerales	Glomeraceae	*Funneliformis*	1	*Funneliformis_mosseae*	1
DG_Otu10	Carrot	296	Glomeromycetes	Diversisporales	Diversisporaceae	*Diversispora*	1	*Diversispora_sp_W2423*	1
DG_Otu11	Carrot	3	Glomeromycetes	Glomerales	Glomeraceae	*Funneliformis*	1	*Funneliformis_mosseae*	0.99
DG_Otu110	Carrot	39	Glomeromycetes	Claroideoglomusles	Claroideoglomeraceae	*Claroideoglomus*	1	*Claroideoglomus_etunicatum*	0.56
DG_Otu12	Carrot	813	Glomeromycetes	Diversisporales	Diversisporaceae	*Diversispora*	1	*Diversispora_celata*	0.99
DG_Otu13	Carrot	880	Glomeromycetes	Claroideoglomusles	Claroideoglomeraceae	*Claroideoglomus*	1	*Glomeromycotina_sp_MIB_8381*	0.93
DG_Otu14	Carrot	1295	Glomeromycetes	Glomerales	Glomeraceae	*Rhizophagus*	1	*Rhizophagus_irregularis*	0.55
DG_Otu15	Carrot	1443	Glomeromycetes	Glomerales	Glomeraceae	*Rhizophagus*	1	*Rhizophagus_irregularis*	0.97
DG_Otu16	Carrot	656	Glomeromycetes	Diversisporales	Diversisporaceae	*Diversispora*	1	*Diversispora_celata*	0.97
DG_Otu17	Carrot	25	Glomeromycetes	Diversisporales	Diversisporaceae	*Diversispora*	1	*Diversispora_sp_W2423*	0.9

DG_Otu18	Carrot	1163	Glomeromycetes	Claroideoglomusles	Claroideoglomeraceae	*Claroideoglomus*	1	*Glomeromycotina_sp_MIB_8381*	1
DG_Otu19	Carrot	176	Glomeromycetes	Claroideoglomusles	Claroideoglomeraceae	*Claroideoglomus*	0.97	*Glomus_sp_NBR_PP1*	0.95
DG_Otu2	Carrot	5313	Glomeromycetes	Glomerales	Glomeraceae	*Rhizophagus*	1	*Glomus_sp_MUCL_43194*	0.55
DG_Otu20	Carrot	34	Glomeromycetes	Claroideoglomusles	Claroideoglomeraceae	*Claroideoglomus*	0.9	*Glomus_sp_NBR_PP1*	0.85
DG_Otu21	Carrot	72	Glomeromycetes	Claroideoglomusles	Claroideoglomeraceae	*Claroideoglomus*	0.93	*Glomus_sp_NBR_PP1*	0.91
DG_Otu3	Carrot	3378	Glomeromycetes	Glomerales	Glomeraceae	*Rhizophagus*	1	*Rhizophagus_irregularis*	0.96
DG_Otu30	Carrot	72	Glomeromycetes	Claroideoglomusles	Claroideoglomeraceae	*Claroideoglomus*	1	*Claroideoglomus_etunicatum*	0.32
DG_Otu4	Carrot	4581	Glomeromycetes	Glomerales	Glomeraceae	*Rhizophagus*	1	*Rhizophagus_irregularis*	0.86
DG_Otu5	Carrot	2904	Glomeromycetes	Claroideoglomusles	Claroideoglomeraceae	*Claroideoglomus*	1	*Glomeromycotina_sp_MIB_8381*	0.58
DG_Otu6	Carrot	5056	Glomeromycetes	Claroideoglomusles	Claroideoglomeraceae	*Claroideoglomus*	0.94	*Claroideoglomus_etunicatum*	0.66
DG_Otu7	Carrot	3175	Glomeromycetes	Glomerales	Glomeraceae	*Rhizophagus*	1	*Rhizophagus_irregularis*	0.64
DG_Otu9	Carrot	1728	Glomeromycetes	Claroideoglomusles	Claroideoglomeraceae	*Claroideoglomus*	0.99	*Glomeromycotina_sp_MIB_8381*	0.55
DG_Otu1	Carrot	155	Glomeromycetes	Glomerales	Glomeraceae	*Funneliformis*	1	*Funneliformis_mosseae*	1
DG_Otu13	Carrot	3899	Glomeromycetes	Claroideoglomusles	Claroideoglomeraceae	*Claroideoglomus*	1	*Glomeromycotina_sp_MIB_8381*	0.93
DG_Otu14	Carrot	255	Glomeromycetes	Glomerales	Glomeraceae	*Rhizophagus*	1	*Rhizophagus_irregularis*	0.55
DG_Otu17	Carrot	29	Glomeromycetes	Diversisporales	Diversisporaceae	*Diversispora*	1	*Diversispora_sp_W2423*	0.9
DG_Otu19	Carrot	193	Glomeromycetes	Claroideoglomusles	Claroideoglomeraceae	*Claroideoglomus*	0.97	*Glomus_sp_NBR_PP1*	0.95
DG_Otu2	Carrot	3467	Glomeromycetes	Glomerales	Glomeraceae	*Rhizophagus*	1	*Glomus_sp_MUCL_43194*	0.55
DG_Otu20	Carrot	35	Glomeromycetes	Claroideoglomusles	Claroideoglomeraceae	*Claroideoglomus*	0.9	*Glomus_sp_NBR_PP1*	0.85
DG_Otu21	Carrot	472	Glomeromycetes	Claroideoglomusles	Claroideoglomeraceae	*Claroideoglomus*	0.93	*Glomus_sp_NBR_PP1*	0.91
DG_Otu26	Carrot	10	Glomeromycetes	Claroideoglomusles	Claroideoglomeraceae	*Claroideoglomus*	0.95	*Glomus_sp_NBR_PP1*	0.93
DG_Otu28	Carrot	181	Glomeromycetes	Claroideoglomusles	Claroideoglomeraceae	*Claroideoglomus*	0.98	*Glomus_sp_NBR_PP1*	0.97
DG_Otu3	Carrot	162	Glomeromycetes	Glomerales	Glomeraceae	*Rhizophagus*	1	*Rhizophagus_irregularis*	0.96
DG_Otu4	Carrot	635	Glomeromycetes	Glomerales	Glomeraceae	*Rhizophagus*	1	*Rhizophagus_irregularis*	0.86
DG_Otu5	Carrot	9692	Glomeromycetes	Claroideoglomusles	Claroideoglomeraceae	*Claroideoglomus*	1	*Glomeromycotina_sp_MIB_8381*	0.58
DG_Otu6	Carrot	6055	Glomeromycetes	Claroideoglomusles	Claroideoglomeraceae	*Claroideoglomus*	0.94	*Claroideoglomus_etunicatum*	0.66
DG_Otu7	Carrot	339	Glomeromycetes	Glomerales	Glomeraceae	*Rhizophagus*	1	*Rhizophagus_irregularis*	0.64
DG_Otu78	Carrot	47	Glomeromycetes	Claroideoglomusles	Claroideoglomeraceae	*Claroideoglomus*	0.99	*Glomus_sp_NBR_PP1*	0.99
DG_Otu9	Carrot	2781	Glomeromycetes	Claroideoglomusles	Claroideoglomeraceae	*Claroideoglomus*	0.99	*Glomeromycotina_sp_MIB_8381*	0.55
DG_Otu1	Onion	####	Glomeromycetes	Glomerales	Glomeraceae	*Funneliformis*	1	*Funneliformis_mosseae*	1
DG_Otu10	Onion	132	Glomeromycetes	Diversisporales	Diversisporaceae	*Diversispora*	1	*Diversispora_sp_W2423*	1
DG_Otu11	Onion	5600	Glomeromycetes	Glomerales	Glomeraceae	*Funneliformis*	1	*Funneliformis_mosseae*	0.99
DG_Otu16	Onion	37	Glomeromycetes	Diversisporales	Diversisporaceae	*Diversispora*	1	*Diversispora_celata*	0.97
DG_Otu2	Onion	128	Glomeromycetes	Glomerales	Glomeraceae	*Rhizophagus*	1	*Glomus_sp_MUCL_43194*	0.55
DG_Otu3	Onion	2345	Glomeromycetes	Glomerales	Glomeraceae	*Rhizophagus*	1	*Rhizophagus_irregularis*	0.96
DG_Otu1	Onion	379	Glomeromycetes	Glomerales	Glomeraceae	*Funneliformis*	1	*Funneliformis_mosseae*	1
DG_Otu10	Onion	2592	Glomeromycetes	Diversisporales	Diversisporaceae	*Diversispora*	1	*Diversispora_sp_W2423*	1
DG_Otu11	Onion	4	Glomeromycetes	Glomerales	Glomeraceae	*Funneliformis*	1	*Funneliformis_mosseae*	0.99
DG_Otu12	Onion	2068	Glomeromycetes	Diversisporales	Diversisporaceae	*Diversispora*	1	*Diversispora_celata*	0.99
DG_Otu16	Onion	272	Glomeromycetes	Diversisporales	Diversisporaceae	*Diversispora*	1	*Diversispora_celata*	0.97
DG_Otu17	Onion	324	Glomeromycetes	Diversisporales	Diversisporaceae	*Diversispora*	1	*Diversispora_sp_W2423*	0.9

DG_Otu19	Onion	265	Glomeromycetes	Claroideoglomusles	Claroideoglomeraceae	*Claroideoglomus*	0.97	*Glomus_sp_NBR_PP1*	0.95
DG_Otu2	Onion	17	Glomeromycetes	Glomerales	Glomeraceae	*Rhizophagus*	1	*Glomus_sp_MUCL_43194*	0.55
DG_Otu3	Onion	####	Glomeromycetes	Glomerales	Glomeraceae	*Rhizophagus*	1	*Rhizophagus_irregularis*	0.96
DG_Otu1	Onion	5356	Glomeromycetes	Glomerales	Glomeraceae	*Funneliformis*	1	*Funneliformis_mosseae*	1
DG_Otu10	Onion	8045	Glomeromycetes	Diversisporales	Diversisporaceae	*Diversispora*	1	*Diversispora_sp_W2423*	1
DG_Otu11	Onion	343	Glomeromycetes	Glomerales	Glomeraceae	*Funneliformis*	1	*Funneliformis_mosseae*	0.99
DG_Otu12	Onion	5247	Glomeromycetes	Diversisporales	Diversisporaceae	*Diversispora*	1	*Diversispora_celata*	0.99
DG_Otu14	Onion	665	Glomeromycetes	Glomerales	Glomeraceae	*Rhizophagus*	1	*Rhizophagus_irregularis*	0.55
DG_Otu15	Onion	2011	Glomeromycetes	Glomerales	Glomeraceae	*Rhizophagus*	1	*Rhizophagus_irregularis*	0.97
DG_Otu16	Onion	1127	Glomeromycetes	Diversisporales	Diversisporaceae	*Diversispora*	1	*Diversispora_celata*	0.97
DG_Otu17	Onion	733	Glomeromycetes	Diversisporales	Diversisporaceae	*Diversispora*	1	*Diversispora_sp_W2423*	0.9
DG_Otu19	Onion	110	Glomeromycetes	Claroideoglomusles	Claroideoglomeraceae	*Claroideoglomus*	0.97	*Glomus_sp_NBR_PP1*	0.95
DG_Otu2	Onion	2387	Glomeromycetes	Glomerales	Glomeraceae	*Rhizophagus*	1	*Glomus_sp_MUCL_43194*	0.55
DG_Otu3	Onion	1389	Glomeromycetes	Glomerales	Glomeraceae	*Rhizophagus*	1	*Rhizophagus_irregularis*	0.96
DG_Otu30	Onion	89	Glomeromycetes	Claroideoglomusles	Claroideoglomeraceae	*Claroideoglomus*	1	*Claroideoglomus_etunicatum*	0.32
DG_Otu37	Onion	32	Glomeromycetes	Claroideoglomusles	Claroideoglomeraceae	*Claroideoglomus*	0.94	*Glomus_sp_NBR_PP1*	0.92
DG_Otu4	Onion	9103	Glomeromycetes	Glomerales	Glomeraceae	*Rhizophagus*	1	*Rhizophagus_irregularis*	0.86
DG_Otu44	Onion	34	Glomeromycetes	Claroideoglomusles	Claroideoglomeraceae	*Claroideoglomus*	0.99	*Glomus_sp_NBR_PP1*	0.96
DG_Otu46	Onion	46	Glomeromycetes	Claroideoglomusles	Claroideoglomeraceae	*Claroideoglomus*	0.95	*Glomus_sp_NBR_PP1*	0.89
DG_Otu5	Onion	100	Glomeromycetes	Claroideoglomusles	Claroideoglomeraceae	*Claroideoglomus*	1	*Glomeromycotina_sp_MIB_8381*	0.58
DG_Otu6	Onion	335	Glomeromycetes	Claroideoglomusles	Claroideoglomeraceae	*Claroideoglomus*	0.94	*Claroideoglomus_etunicatum*	0.66
DG_Otu7	Onion	8484	Glomeromycetes	Glomerales	Glomeraceae	*Rhizophagus*	1	*Rhizophagus_irregularis*	0.64
DG_Otu9	Onion	87	Glomeromycetes	Claroideoglomusles	Claroideoglomeraceae	*Claroideoglomus*	0.99	*Glomeromycotina_sp_MIB_8381*	0.55
DG_Otu1	Onion	####	Glomeromycetes	Glomerales	Glomeraceae	*Funneliformis*	1	*Funneliformis_mosseae*	1
DG_Otu10	Onion	2471	Glomeromycetes	Diversisporales	Diversisporaceae	*Diversispora*	1	*Diversispora_sp_W2423*	1
DG_Otu11	Onion	999	Glomeromycetes	Glomerales	Glomeraceae	*Funneliformis*	1	*Funneliformis_mosseae*	0.99
DG_Otu12	Onion	7045	Glomeromycetes	Diversisporales	Diversisporaceae	*Diversispora*	1	*Diversispora_celata*	0.99
DG_Otu13	Onion	53	Glomeromycetes	Claroideoglomusles	Claroideoglomeraceae	*Claroideoglomus*	1	*Glomeromycotina_sp_MIB_8381*	0.93
DG_Otu14	Onion	913	Glomeromycetes	Glomerales	Glomeraceae	*Rhizophagus*	1	*Rhizophagus_irregularis*	0.55
DG_Otu15	Onion	133	Glomeromycetes	Glomerales	Glomeraceae	*Rhizophagus*	1	*Rhizophagus_irregularis*	0.97
DG_Otu16	Onion	1801	Glomeromycetes	Diversisporales	Diversisporaceae	*Diversispora*	1	*Diversispora_celata*	0.97
DG_Otu17	Onion	189	Glomeromycetes	Diversisporales	Diversisporaceae	*Diversispora*	1	*Diversispora_sp_W2423*	0.9
DG_Otu18	Onion	47	Glomeromycetes	Claroideoglomusles	Claroideoglomeraceae	*Claroideoglomus*	1	*Glomeromycotina_sp_MIB_8381*	1
DG_Otu2	Onion	281	Glomeromycetes	Glomerales	Glomeraceae	*Rhizophagus*	1	*Glomus_sp_MUCL_43194*	0.55
DG_Otu20	Onion	145	Glomeromycetes	Claroideoglomusles	Claroideoglomeraceae	*Claroideoglomus*	0.9	*Glomus_sp_NBR_PP1*	0.85
DG_Otu3	Onion	432	Glomeromycetes	Glomerales	Glomeraceae	*Rhizophagus*	1	*Rhizophagus_irregularis*	0.96
DG_Otu4	Onion	3160	Glomeromycetes	Glomerales	Glomeraceae	*Rhizophagus*	1	*Rhizophagus_irregularis*	0.86
DG_Otu46	Onion	50	Glomeromycetes	Claroideoglomusles	Claroideoglomeraceae	*Claroideoglomus*	0.95	*Glomus_sp_NBR_PP1*	0.89
DG_Otu5	Onion	172	Glomeromycetes	Claroideoglomusles	Claroideoglomeraceae	*Claroideoglomus*	1	*Glomeromycotina_sp_MIB_8381*	0.58
DG_Otu6	Onion	186	Glomeromycetes	Claroideoglomusles	Claroideoglomeraceae	*Claroideoglomus*	0.94	*Claroideoglomus_etunicatum*	0.66
DG_Otu7	Onion	1526	Glomeromycetes	Glomerales	Glomeraceae	*Rhizophagus*	1	*Rhizophagus_irregularis*	0.64

DG_Otu1	Onion	####	Glomeromycetes	Glomerales	Glomeraceae	*Funneliformis*	1	*Funneliformis_mosseae*	1
DG_Otu10	Onion	620	Glomeromycetes	Diversisporales	Diversisporaceae	*Diversispora*	1	*Diversispora_sp_W2423*	1
DG_Otu11	Onion	8373	Glomeromycetes	Glomerales	Glomeraceae	*Funneliformis*	1	*Funneliformis_mosseae*	0.99
DG_Otu2	Onion	1403	Glomeromycetes	Glomerales	Glomeraceae	*Rhizophagus*	1	*Glomus_sp_MUCL_43194*	0.55
DG_Otu3	Onion	6228	Glomeromycetes	Glomerales	Glomeraceae	*Rhizophagus*	1	*Rhizophagus_irregularis*	0.96
DG_Otu7	Onion	4	Glomeromycetes	Glomerales	Glomeraceae	*Rhizophagus*	1	*Rhizophagus_irregularis*	0.64
DG_Otu1	Onion	886	Glomeromycetes	Glomerales	Glomeraceae	*Funneliformis*	1	*Funneliformis_mosseae*	1
DG_Otu11	Onion	3	Glomeromycetes	Glomerales	Glomeraceae	*Funneliformis*	1	*Funneliformis_mosseae*	0.99
DG_Otu14	Onion	23	Glomeromycetes	Glomerales	Glomeraceae	*Rhizophagus*	1	*Rhizophagus_irregularis*	0.55
DG_Otu2	Onion	5	Glomeromycetes	Glomerales	Glomeraceae	*Rhizophagus*	1	*Glomus_sp_MUCL_43194*	0.55
DG_Otu3	Onion	38	Glomeromycetes	Glomerales	Glomeraceae	*Rhizophagus*	1	*Rhizophagus_irregularis*	0.96
DG_Otu4	Onion	50	Glomeromycetes	Glomerales	Glomeraceae	*Rhizophagus*	1	*Rhizophagus_irregularis*	0.86
DG_Otu5	Onion	3	Glomeromycetes	Claroideoglomusles	Claroideoglomeraceae	*Claroideoglomus*	1	*Glomeromycotina_sp_MIB_8381*	0.58
DG_Otu7	Onion	38	Glomeromycetes	Glomerales	Glomeraceae	*Rhizophagus*	1	*Rhizophagus_irregularis*	0.64
DG_Otu1	Onion	8345	Glomeromycetes	Glomerales	Glomeraceae	*Funneliformis*	1	*Funneliformis_mosseae*	1
DG_Otu10	Onion	1896	Glomeromycetes	Diversisporales	Diversisporaceae	*Diversispora*	1	*Diversispora_sp_W2423*	1
DG_Otu106	Onion	3	Glomeromycetes	Glomerales	Glomeraceae	*Funneliformis*	0.96	*Funneliformis_mosseae*	0.89
DG_Otu11	Onion	1015	Glomeromycetes	Glomerales	Glomeraceae	*Funneliformis*	1	*Funneliformis_mosseae*	0.99
DG_Otu12	Onion	4034	Glomeromycetes	Diversisporales	Diversisporaceae	*Diversispora*	1	*Diversispora_celata*	0.99
DG_Otu13	Onion	512	Glomeromycetes	Claroideoglomusles	Claroideoglomeraceae	*Claroideoglomus*	1	*Glomeromycotina_sp_MIB_8381*	0.93
DG_Otu14	Onion	1030	Glomeromycetes	Glomerales	Glomeraceae	*Rhizophagus*	1	*Rhizophagus_irregularis*	0.55
DG_Otu15	Onion	1623	Glomeromycetes	Glomerales	Glomeraceae	*Rhizophagus*	1	*Rhizophagus_irregularis*	0.97
DG_Otu16	Onion	2817	Glomeromycetes	Diversisporales	Diversisporaceae	*Diversispora*	1	*Diversispora_celata*	0.97
DG_Otu17	Onion	132	Glomeromycetes	Diversisporales	Diversisporaceae	*Diversispora*	1	*Diversispora_sp_W2423*	0.9
DG_Otu2	Onion	3156	Glomeromycetes	Glomerales	Glomeraceae	*Rhizophagus*	1	*Glomus_sp_MUCL_43194*	0.55
DG_Otu3	Onion	1875	Glomeromycetes	Glomerales	Glomeraceae	*Rhizophagus*	1	*Rhizophagus_irregularis*	0.96
DG_Otu4	Onion	8595	Glomeromycetes	Glomerales	Glomeraceae	*Rhizophagus*	1	*Rhizophagus_irregularis*	0.86
DG_Otu5	Onion	2792	Glomeromycetes	Claroideoglomusles	Claroideoglomeraceae	*Claroideoglomus*	1	*Glomeromycotina_sp_MIB_8381*	0.58
DG_Otu6	Onion	175	Glomeromycetes	Claroideoglomusles	Claroideoglomeraceae	*Claroideoglomus*	0.94	*Claroideoglomus_etunicatum*	0.66
DG_Otu7	Onion	4511	Glomeromycetes	Glomerales	Glomeraceae	*Rhizophagus*	1	*Rhizophagus_irregularis*	0.64
DG_Otu9	Onion	782	Glomeromycetes	Claroideoglomusles	Claroideoglomeraceae	*Claroideoglomus*	0.99	*Glomeromycotina_sp_MIB_8381*	0.55
DG_Otu95	Onion	16	Glomeromycetes	Glomerales	Glomeraceae	*Funneliformis*	0.99	*Funneliformis_mosseae*	0.97
DG_Otu1	Onion	1684	Glomeromycetes	Glomerales	Glomeraceae	*Funneliformis*	1	*Funneliformis_mosseae*	1
DG_Otu10	Onion	358	Glomeromycetes	Diversisporales	Diversisporaceae	*Diversispora*	1	*Diversispora_sp_W2423*	1
DG_Otu11	Onion	170	Glomeromycetes	Glomerales	Glomeraceae	*Funneliformis*	1	*Funneliformis_mosseae*	0.99
DG_Otu12	Onion	391	Glomeromycetes	Diversisporales	Diversisporaceae	*Diversispora*	1	*Diversispora_celata*	0.99
DG_Otu14	Onion	33	Glomeromycetes	Glomerales	Glomeraceae	*Rhizophagus*	1	*Rhizophagus_irregularis*	0.55
DG_Otu15	Onion	104	Glomeromycetes	Glomerales	Glomeraceae	*Rhizophagus*	1	*Rhizophagus_irregularis*	0.97
DG_Otu16	Onion	353	Glomeromycetes	Diversisporales	Diversisporaceae	*Diversispora*	1	*Diversispora_celata*	0.97
DG_Otu17	Onion	23	Glomeromycetes	Diversisporales	Diversisporaceae	*Diversispora*	1	*Diversispora_sp_W2423*	0.9
DG_Otu18	Onion	131	Glomeromycetes	Claroideoglomusles	Claroideoglomeraceae	*Claroideoglomus*	1	*Glomeromycotina_sp_MIB_8381*	1

DG_Otu19	Onion	6	Glomeromycetes	Claroideoglomusles	Claroideoglomeraceae	*Claroideoglomus*	0.97	*Glomus_sp_NBR_PP1*	0.95
DG_Otu2	Onion	3004	Glomeromycetes	Glomerales	Glomeraceae	*Rhizophagus*	1	*Glomus_sp_MUCL_43194*	0.55
DG_Otu21	Onion	100	Glomeromycetes	Claroideoglomusles	Claroideoglomeraceae	*Claroideoglomus*	0.93	*Glomus_sp_NBR_PP1*	0.91
DG_Otu26	Onion	41	Glomeromycetes	Claroideoglomusles	Claroideoglomeraceae	*Claroideoglomus*	0.95	*Glomus_sp_NBR_PP1*	0.93
DG_Otu28	Onion	10	Glomeromycetes	Claroideoglomusles	Claroideoglomeraceae	*Claroideoglomus*	0.98	*Glomus_sp_NBR_PP1*	0.97
DG_Otu3	Onion	923	Glomeromycetes	Glomerales	Glomeraceae	*Rhizophagus*	1	*Rhizophagus_irregularis*	0.96
DG_Otu30	Onion	21	Glomeromycetes	Claroideoglomusles	Claroideoglomeraceae	*Claroideoglomus*	1	*Claroideoglomus_etunicatum*	0.32
DG_Otu4	Onion	####	Glomeromycetes	Glomerales	Glomeraceae	*Rhizophagus*	1	*Rhizophagus_irregularis*	0.86
DG_Otu43	Onion	4	Glomeromycetes	Glomerales	Glomerales_uncultured	*Glomerales_undefined*	0.92	*Glomeromycotina_sp_8536*	0.81
DG_Otu5	Onion	18	Glomeromycetes	Claroideoglomusles	Claroideoglomeraceae	*Claroideoglomus*	1	*Glomeromycotina_sp_MIB_8381*	0.58
DG_Otu6	Onion	2967	Glomeromycetes	Claroideoglomusles	Claroideoglomeraceae	*Claroideoglomus*	0.94	*Claroideoglomus_etunicatum*	0.66
DG_Otu7	Onion	8038	Glomeromycetes	Glomerales	Glomeraceae	*Rhizophagus*	1	*Rhizophagus_irregularis*	0.64
DG_Otu9	Onion	5	Glomeromycetes	Claroideoglomusles	Claroideoglomeraceae	*Claroideoglomus*	0.99	*Glomeromycotina_sp_MIB_8381*	0.55
DG_Otu1	Onion	####	Glomeromycetes	Glomerales	Glomeraceae	*Funneliformis*	1	*Funneliformis_mosseae*	1
DG_Otu11	Onion	7818	Glomeromycetes	Glomerales	Glomeraceae	*Funneliformis*	1	*Funneliformis_mosseae*	0.99
DG_Otu2	Onion	504	Glomeromycetes	Glomerales	Glomeraceae	*Rhizophagus*	1	*Glomus_sp_MUCL_43194*	0.55
DG_Otu3	Onion	3239	Glomeromycetes	Glomerales	Glomeraceae	*Rhizophagus*	1	*Rhizophagus_irregularis*	0.96
DG_Otu1	Onion	2091	Glomeromycetes	Glomerales	Glomeraceae	*Funneliformis*	1	*Funneliformis_mosseae*	1
DG_Otu10	Onion	433	Glomeromycetes	Diversisporales	Diversisporaceae	*Diversispora*	1	*Diversispora_sp_W2423*	1
DG_Otu11	Onion	6	Glomeromycetes	Glomerales	Glomeraceae	*Funneliformis*	1	*Funneliformis_mosseae*	0.99
DG_Otu12	Onion	192	Glomeromycetes	Diversisporales	Diversisporaceae	*Diversispora*	1	*Diversispora_celata*	0.99
DG_Otu14	Onion	80	Glomeromycetes	Glomerales	Glomeraceae	*Rhizophagus*	1	*Rhizophagus_irregularis*	0.55
DG_Otu16	Onion	50	Glomeromycetes	Diversisporales	Diversisporaceae	*Diversispora*	1	*Diversispora_celata*	0.97
DG_Otu2	Onion	6	Glomeromycetes	Glomerales	Glomeraceae	*Rhizophagus*	1	*Glomus_sp_MUCL_43194*	0.55
DG_Otu3	Onion	3692	Glomeromycetes	Glomerales	Glomeraceae	*Rhizophagus*	1	*Rhizophagus_irregularis*	0.96
DG_Otu4	Onion	25	Glomeromycetes	Glomerales	Glomeraceae	*Rhizophagus*	1	*Rhizophagus_irregularis*	0.86
DG_Otu1	Onion	3183	Glomeromycetes	Glomerales	Glomeraceae	*Funneliformis*	1	*Funneliformis_mosseae*	1
DG_Otu10	Onion	7438	Glomeromycetes	Diversisporales	Diversisporaceae	*Diversispora*	1	*Diversispora_sp_W2423*	1
DG_Otu11	Onion	67	Glomeromycetes	Glomerales	Glomeraceae	*Funneliformis*	1	*Funneliformis_mosseae*	0.99
DG_Otu12	Onion	1966	Glomeromycetes	Diversisporales	Diversisporaceae	*Diversispora*	1	*Diversispora_celata*	0.99
DG_Otu14	Onion	197	Glomeromycetes	Glomerales	Glomeraceae	*Rhizophagus*	1	*Rhizophagus_irregularis*	0.55
DG_Otu15	Onion	296	Glomeromycetes	Glomerales	Glomeraceae	*Rhizophagus*	1	*Rhizophagus_irregularis*	0.97
DG_Otu16	Onion	1660	Glomeromycetes	Diversisporales	Diversisporaceae	*Diversispora*	1	*Diversispora_celata*	0.97
DG_Otu17	Onion	1105	Glomeromycetes	Diversisporales	Diversisporaceae	*Diversispora*	1	*Diversispora_sp_W2423*	0.9
DG_Otu2	Onion	1023	Glomeromycetes	Glomerales	Glomeraceae	*Rhizophagus*	1	*Glomus_sp_MUCL_43194*	0.55
DG_Otu3	Onion	315	Glomeromycetes	Glomerales	Glomeraceae	*Rhizophagus*	1	*Rhizophagus_irregularis*	0.96
DG_Otu4	Onion	1161	Glomeromycetes	Glomerales	Glomeraceae	*Rhizophagus*	1	*Rhizophagus_irregularis*	0.86
DG_Otu43	Onion	57	Glomeromycetes	Glomerales	Glomerales_uncultured	*Glomerales_undefined*	0.92	*Glomeromycotina_sp_8536*	0.81
DG_Otu47	Onion	39	Glomeromycetes	Glomerales	Glomerales_uncultured	*Glomerales_undefined*	0.97	*Glomeromycotina_sp_8536*	0.96
DG_Otu6	Onion	67	Glomeromycetes	Claroideoglomusles	Claroideoglomeraceae	*Claroideoglomus*	0.94	*Claroideoglomus_etunicatum*	0.66
DG_Otu7	Onion	1088	Glomeromycetes	Glomerales	Glomeraceae	*Rhizophagus*	1	*Rhizophagus_irregularis*	0.64

DG_Otu80	Onion	67	Glomeromycetes	Glomerales	Glomerales_uncultured	*Glomerales_undefined*	0.94	*Glomeromycotina_sp_8536*	0.92
DG_Otu94	Onion	12	Glomeromycetes	Glomerales	Glomerales_uncultured	*Glomerales_undefined*	0.94	*Glomeromycotina_sp_8536*	0.91
DG_Otu1	Onion	####	Glomeromycetes	Glomerales	Glomeraceae	*Funneliformis*	1	*Funneliformis_mosseae*	1
DG_Otu10	Onion	264	Glomeromycetes	Diversisporales	Diversisporaceae	*Diversispora*	1	*Diversispora_sp_W2423*	1
DG_Otu100	Onion	47	Glomeromycetes	Claroideoglomusles	Claroideoglomeraceae	*Claroideoglomus*	1	*Glomus_sp_NBR31*	0.29
DG_Otu106	Onion	36	Glomeromycetes	Glomerales	Glomeraceae	*Funneliformis*	0.96	*Funneliformis_mosseae*	0.89
DG_Otu11	Onion	1363	Glomeromycetes	Glomerales	Glomeraceae	*Funneliformis*	1	*Funneliformis_mosseae*	0.99
DG_Otu12	Onion	584	Glomeromycetes	Diversisporales	Diversisporaceae	*Diversispora*	1	*Diversispora_celata*	0.99
DG_Otu13	Onion	152	Glomeromycetes	Claroideoglomusles	Claroideoglomeraceae	*Claroideoglomus*	1	*Glomeromycotina_sp_MIB_8381*	0.93
DG_Otu14	Onion	153	Glomeromycetes	Glomerales	Glomeraceae	*Rhizophagus*	1	*Rhizophagus_irregularis*	0.55
DG_Otu15	Onion	86	Glomeromycetes	Glomerales	Glomeraceae	*Rhizophagus*	1	*Rhizophagus_irregularis*	0.97
DG_Otu154	Onion	3	Glomeromycetes	Glomerales	Glomeraceae	*Funneliformis*	1	*Funneliformis_mosseae*	1
DG_Otu16	Onion	109	Glomeromycetes	Diversisporales	Diversisporaceae	*Diversispora*	1	*Diversispora_celata*	0.97
DG_Otu17	Onion	6	Glomeromycetes	Diversisporales	Diversisporaceae	*Diversispora*	1	*Diversispora_sp_W2423*	0.9
DG_Otu19	Onion	82	Glomeromycetes	Claroideoglomusles	Claroideoglomeraceae	*Claroideoglomus*	0.97	*Glomus_sp_NBR_PP1*	0.95
DG_Otu2	Onion	####	Glomeromycetes	Glomerales	Glomeraceae	*Rhizophagus*	1	*Glomus_sp_MUCL_43194*	0.55
DG_Otu3	Onion	4775	Glomeromycetes	Glomerales	Glomeraceae	*Rhizophagus*	1	*Rhizophagus_irregularis*	0.96
DG_Otu30	Onion	144	Glomeromycetes	Claroideoglomusles	Claroideoglomeraceae	*Claroideoglomus*	1	*Claroideoglomus_etunicatum*	0.32
DG_Otu4	Onion	357	Glomeromycetes	Glomerales	Glomeraceae	*Rhizophagus*	1	*Rhizophagus_irregularis*	0.86
DG_Otu5	Onion	633	Glomeromycetes	Claroideoglomusles	Claroideoglomeraceae	*Claroideoglomus*	1	*Glomeromycotina_sp_MIB_8381*	0.58
DG_Otu58	Onion	30	Glomeromycetes	Claroideoglomusles	Claroideoglomeraceae	*Claroideoglomus*	0.99	*Glomus_sp_NBR_PP1*	0.99
DG_Otu6	Onion	690	Glomeromycetes	Claroideoglomusles	Claroideoglomeraceae	*Claroideoglomus*	0.94	*Claroideoglomus_etunicatum*	0.66
DG_Otu7	Onion	442	Glomeromycetes	Glomerales	Glomeraceae	*Rhizophagus*	1	*Rhizophagus_irregularis*	0.64
DG_Otu9	Onion	56	Glomeromycetes	Claroideoglomusles	Claroideoglomeraceae	*Claroideoglomus*	0.99	*Glomeromycotina_sp_MIB_8381*	0.55
DG_Otu95	Onion	32	Glomeromycetes	Glomerales	Glomeraceae	*Funneliformis*	0.99	*Funneliformis_mosseae*	0.97
DG_Otu1	Onion	####	Glomeromycetes	Glomerales	Glomeraceae	*Funneliformis*	1	*Funneliformis_mosseae*	1
DG_Otu10	Onion	721	Glomeromycetes	Diversisporales	Diversisporaceae	*Diversispora*	1	*Diversispora_sp_W2423*	1
DG_Otu11	Onion	5231	Glomeromycetes	Glomerales	Glomeraceae	*Funneliformis*	1	*Funneliformis_mosseae*	0.99
DG_Otu12	Onion	76	Glomeromycetes	Diversisporales	Diversisporaceae	*Diversispora*	1	*Diversispora_celata*	0.99
DG_Otu2	Onion	596	Glomeromycetes	Glomerales	Glomeraceae	*Rhizophagus*	1	*Glomus_sp_MUCL_43194*	0.55
DG_Otu3	Onion	2074	Glomeromycetes	Glomerales	Glomeraceae	*Rhizophagus*	1	*Rhizophagus_irregularis*	0.96
DG_Otu1	Onion	8899	Glomeromycetes	Glomerales	Glomeraceae	*Funneliformis*	1	*Funneliformis_mosseae*	1
DG_Otu10	Onion	1079	Glomeromycetes	Diversisporales	Diversisporaceae	*Diversispora*	1	*Diversispora_sp_W2423*	1
DG_Otu11	Onion	28	Glomeromycetes	Glomerales	Glomeraceae	*Funneliformis*	1	*Funneliformis_mosseae*	0.99
DG_Otu12	Onion	1012	Glomeromycetes	Diversisporales	Diversisporaceae	*Diversispora*	1	*Diversispora_celata*	0.99
DG_Otu14	Onion	669	Glomeromycetes	Glomerales	Glomeraceae	*Rhizophagus*	1	*Rhizophagus_irregularis*	0.55
DG_Otu16	Onion	774	Glomeromycetes	Diversisporales	Diversisporaceae	*Diversispora*	1	*Diversispora_celata*	0.97
DG_Otu17	Onion	520	Glomeromycetes	Diversisporales	Diversisporaceae	*Diversispora*	1	*Diversispora_sp_W2423*	0.9
DG_Otu2	Onion	17	Glomeromycetes	Glomerales	Glomeraceae	*Rhizophagus*	1	*Glomus_sp_MUCL_43194*	0.55
DG_Otu3	Onion	####	Glomeromycetes	Glomerales	Glomeraceae	*Rhizophagus*	1	*Rhizophagus_irregularis*	0.96
DG_Otu4	Onion	607	Glomeromycetes	Glomerales	Glomeraceae	*Rhizophagus*	1	*Rhizophagus_irregularis*	0.86

DG_Otu5	Onion	3	Glomeromycetes	Claroideoglomusles	Claroideoglomeraceae	*Claroideoglomus*	1	*Glomeromycotina_sp_MIB_8381*	0.58
DG_Otu7	Onion	436	Glomeromycetes	Glomerales	Glomeraceae	*Rhizophagus*	1	*Rhizophagus_irregularis*	0.64
DG_Otu1	Onion	####	Glomeromycetes	Glomerales	Glomeraceae	*Funneliformis*	1	*Funneliformis_mosseae*	1
DG_Otu10	Onion	4088	Glomeromycetes	Diversisporales	Diversisporaceae	*Diversispora*	1	*Diversispora_sp_W2423*	1
DG_Otu11	Onion	232	Glomeromycetes	Glomerales	Glomeraceae	*Funneliformis*	1	*Funneliformis_mosseae*	0.99
DG_Otu12	Onion	6976	Glomeromycetes	Diversisporales	Diversisporaceae	*Diversispora*	1	*Diversispora_celata*	0.99
DG_Otu14	Onion	592	Glomeromycetes	Glomerales	Glomeraceae	*Rhizophagus*	1	*Rhizophagus_irregularis*	0.55
DG_Otu15	Onion	903	Glomeromycetes	Glomerales	Glomeraceae	*Rhizophagus*	1	*Rhizophagus_irregularis*	0.97
DG_Otu16	Onion	2949	Glomeromycetes	Diversisporales	Diversisporaceae	*Diversispora*	1	*Diversispora_celata*	0.97
DG_Otu17	Onion	1315	Glomeromycetes	Diversisporales	Diversisporaceae	*Diversispora*	1	*Diversispora_sp_W2423*	0.9
DG_Otu18	Onion	64	Glomeromycetes	Claroideoglomusles	Claroideoglomeraceae	*Claroideoglomus*	1	*Glomeromycotina_sp_MIB_8381*	1
DG_Otu2	Onion	1344	Glomeromycetes	Glomerales	Glomeraceae	*Rhizophagus*	1	*Glomus_sp_MUCL_43194*	0.55
DG_Otu3	Onion	6590	Glomeromycetes	Glomerales	Glomeraceae	*Rhizophagus*	1	*Rhizophagus_irregularis*	0.96
DG_Otu37	Onion	73	Glomeromycetes	Claroideoglomusles	Claroideoglomeraceae	*Claroideoglomus*	0.94	*Glomus_sp_NBR_PP1*	0.92
DG_Otu4	Onion	4850	Glomeromycetes	Glomerales	Glomeraceae	*Rhizophagus*	1	*Rhizophagus_irregularis*	0.86
DG_Otu43	Onion	86	Glomeromycetes	Glomerales	Glomerales_uncultured	*Glomerales_undefined*	0.92	*Glomeromycotina_sp_8536*	0.81
DG_Otu44	Onion	95	Glomeromycetes	Claroideoglomusles	Claroideoglomeraceae	*Claroideoglomus*	0.99	*Glomus_sp_NBR_PP1*	0.96
DG_Otu46	Onion	46	Glomeromycetes	Claroideoglomusles	Claroideoglomeraceae	*Claroideoglomus*	0.95	*Glomus_sp_NBR_PP1*	0.89
DG_Otu47	Onion	21	Glomeromycetes	Glomerales	Glomerales_uncultured	*Glomerales_undefined*	0.97	*Glomeromycotina_sp_8536*	0.96
DG_Otu6	Onion	22	Glomeromycetes	Claroideoglomusles	Claroideoglomeraceae	*Claroideoglomus*	0.94	*Claroideoglomus_etunicatum*	0.66
DG_Otu7	Onion	3570	Glomeromycetes	Glomerales	Glomeraceae	*Rhizophagus*	1	*Rhizophagus_irregularis*	0.64
DG_Otu80	Onion	17	Glomeromycetes	Glomerales	Glomerales_uncultured	*Glomerales_undefined*	0.94	*Glomeromycotina_sp_8536*	0.92
DG_Otu94	Onion	45	Glomeromycetes	Glomerales	Glomerales_uncultured	*Glomerales_undefined*	0.94	*Glomeromycotina_sp_8536*	0.91
DG_Otu1	Onion	571	Glomeromycetes	Glomerales	Glomeraceae	*Funneliformis*	1	*Funneliformis_mosseae*	1
DG_Otu10	Onion	1308	Glomeromycetes	Diversisporales	Diversisporaceae	*Diversispora*	1	*Diversispora_sp_W2423*	1
DG_Otu11	Onion	14	Glomeromycetes	Glomerales	Glomeraceae	*Funneliformis*	1	*Funneliformis_mosseae*	0.99
DG_Otu12	Onion	125	Glomeromycetes	Diversisporales	Diversisporaceae	*Diversispora*	1	*Diversispora_celata*	0.99
DG_Otu14	Onion	2549	Glomeromycetes	Glomerales	Glomeraceae	*Rhizophagus*	1	*Rhizophagus_irregularis*	0.55
DG_Otu15	Onion	1170	Glomeromycetes	Glomerales	Glomeraceae	*Rhizophagus*	1	*Rhizophagus_irregularis*	0.97
DG_Otu16	Onion	47	Glomeromycetes	Diversisporales	Diversisporaceae	*Diversispora*	1	*Diversispora_celata*	0.97
DG_Otu17	Onion	101	Glomeromycetes	Diversisporales	Diversisporaceae	*Diversispora*	1	*Diversispora_sp_W2423*	0.9
DG_Otu2	Onion	9832	Glomeromycetes	Glomerales	Glomeraceae	*Rhizophagus*	1	*Glomus_sp_MUCL_43194*	0.55
DG_Otu21	Onion	104	Glomeromycetes	Claroideoglomusles	Claroideoglomeraceae	*Claroideoglomus*	0.93	*Glomus_sp_NBR_PP1*	0.91
DG_Otu26	Onion	63	Glomeromycetes	Claroideoglomusles	Claroideoglomeraceae	*Claroideoglomus*	0.95	*Glomus_sp_NBR_PP1*	0.93
DG_Otu3	Onion	3856	Glomeromycetes	Glomerales	Glomeraceae	*Rhizophagus*	1	*Rhizophagus_irregularis*	0.96
DG_Otu4	Onion	5985	Glomeromycetes	Glomerales	Glomeraceae	*Rhizophagus*	1	*Rhizophagus_irregularis*	0.86
DG_Otu5	Onion	6	Glomeromycetes	Claroideoglomusles	Claroideoglomeraceae	*Claroideoglomus*	1	*Glomeromycotina_sp_MIB_8381*	0.58
DG_Otu6	Onion	348	Glomeromycetes	Claroideoglomusles	Claroideoglomeraceae	*Claroideoglomus*	0.94	*Claroideoglomus_etunicatum*	0.66
DG_Otu7	Onion	3729	Glomeromycetes	Glomerales	Glomeraceae	*Rhizophagus*	1	*Rhizophagus_irregularis*	0.64
DG_Otu95	Onion	3	Glomeromycetes	Glomerales	Glomeraceae	*Funneliformis*	0.99	*Funneliformis_mosseae*	0.97

BP stands for confidence threshold values
