# Supplementary material for: Influence of plant species, mycorrhizal inoculant, and soil phosphorus level on arbuscular mycorrhizal communities in onion and carrot roots
Source: Front Plant Sci. 2024 Jan 15;14:1324626. doi: 10.3389/fpls.2023.1324626 (PMC10823018; doi:10.3389/fpls.2023.1324626)
Supplement: Supplementary file 2 [file Table_2.docx]

**Supplementary Table S2**. Arbuscular mycorrhizal fungi colonizing the roots of onion and carrot plants grown on high organic matter (muck) soil in a field trial in the Holland Marsh, Ontario, Canada

| Order | Family | Genus (bootstrap ≥ 90) | Potential species ^1^ |
| --- | --- | --- | --- |
| Diversiporales | Diversisporaceae | *Diversispora* | *D. celata* |
|  |  |  | *Diversispora* sp. W2423 |
| Entrophosporales | Claroideoglomeraceae^2^ | *Claroideoglomus* | *C. etunicatum* |
|  |  |  |  |
|  |  |  |  |
| Glomerales | Glomeraceae | *Funneliformis* | *F. mosseae* |
|  |  | *Glomus* | *G. macrocarpum* ^3^  Glomeromycotina MIB 8381  *Glomus* sp. NBR PP1  *Glomus* sp. NBR31^4^ |
|  |  | Glomerales undefined | Glomerales undefined |
|  |  | *Rhizophagus* | *Glomus* sp. MUCL 43194 |
|  |  |  | *R. irregularis* ^5^ |

^1^ The confidence threshold ranges from 0 to 100 at the species-level for potential species identified with two sets of primers, AMV 4.5NF - AMDGR, using Illumina MiSeq.

^2^ According to recent phylogenomic analyses by Janusz Błaszkowski et al. 2022, Claroideoglomeraceae has been synonymized with Entrophosporaceae, *Claroideoglomus with Entrophospora*

^3^ Identified only in roots of onion plants, not carrot plants.

^4^ Identified only in roots of onion plants, not carrot plants.

^5^Previously known as *Glomus irregularis*.
