# Supplementary material for: Influence of plant species, mycorrhizal inoculant, and soil phosphorus level on arbuscular mycorrhizal communities in onion and carrot roots
Source: Front Plant Sci. 2024 Jan 15;14:1324626. doi: 10.3389/fpls.2023.1324626 (PMC10823018; doi:10.3389/fpls.2023.1324626)
Supplement: Supplementary file 3 [file Table_3.docx]

**Supplementary Table S3**. Analysis of variance (ANOVA) table for the alpha diversity of arbuscular mycorrhizal fungi colonizing the roots of onions and carrots grown on muck soil in a field trial at Holland Marsh, Ontario

| **Split-split-plot AOV Table** | | | | | | | | | | | | | |
| --- | --- | --- | --- | --- | --- | --- | --- | --- | --- | --- | --- | --- | --- |
|  | | Richness | | | | Shannon`s Index | | | | Inverse Simpson`s index | | | |
| Source of variation | DF | SS | MS | F | *P* | SS | MS | F | *P* | SS | MS | F | *P* |
| block | 3 | 15.344 | 5.115 |  |  | 0.415 | 0.13833 |  |  | 4.516 | 1.5053 |  |  |
| Plant type | 1 | 108.781 | 108.781 | 58.34 | **0.0047** | 2.88 | 2.88 | 42.15 | **0.0074** | 51.765 | 51.7653 | 19.97 | **0.0209** |
| Error block*Plant type | 3 | 5.594 | 1.865 |  |  | 0.205 | 0.06833 |  |  | 7.776 | 2.592 |  |  |
| P | 1 | 0.031 | 0.031 | 0.01 | 0.9421 | 0.1013 | 0.10125 | 1.65 | 0.2459 | 10.928 | 10.9278 | 7.19 | 0.0365 |
| Plant type*P | 1 | 34.031 | 34.031 | 6.25 | **0.0466** | 0.0312 | 0.03125 | 0.51 | 0.5019 | 0.525 | 0.5253 | 0.35 | 0.5781 |
| Error block*Plant type*P | 6 | 32.688 | 5.448 |  |  | 0.3675 | 0.06125 |  |  | 9.124 | 1.5207 |  |  |
| AMF | 1 | 225.781 | 225.781 | 14.14 | 0.0027 | 1.28 | 1.28 | 9.93 | 0.0084 | 14.445 | 14.4453 | 2.96 | 0.1112 |
| Plant type*AMF | 1 | 306.281 | 306.281 | 19.18 | **0.0009** | 3.38 | 3.38 | 26.21 | **0.0003** | 36.338 | 36.3378 | 7.43 | **0.0184** |
| P*AMF | 1 | 0.781 | 0.781 | 0.05 | 0.8287 | 0.0113 | 0.01125 | 0.09 | 0.7728 | 2.153 | 2.1528 | 0.44 | 0.5194 |
| Plant type*P*AMF | 1 | 34.031 | 34.031 | 2.13 | 0.17 | 0.6613 | 0.66125 | 5.13 | 0.0629 | 12.128 | 12.1278 | 2.48 | 0.1412 |
| Error block*plant type*P*AMF | 12 | 191.625 | 15.969 |  |  | 1.5475 | 0.12896 |  |  | 58.651 | 4.8876 |  |  |
| Total | 31 | 954.969 |  |  |  | 10.88 |  |  |  | 208.35 |  |  |  |

Plant type represented onions and carrots. AMF stands for arbuscular mycorrhizal fungal inoculant, and P for soil phosphorus levels.
