# Supplementary material for: Influence of plant species, mycorrhizal inoculant, and soil phosphorus level on arbuscular mycorrhizal communities in onion and carrot roots
Source: Front Plant Sci. 2024 Jan 15;14:1324626. doi: 10.3389/fpls.2023.1324626 (PMC10823018; doi:10.3389/fpls.2023.1324626)
Supplement: Supplementary Figure 1 — Position of binding of the primer pair AMV4.5F – AMDGR (Sato et al., 2005) on the small subunit of the 18S ribosomal RNA gene. The lines represent the region of binding by the specific primers. Arrows on the lines indicate the direction of sequencing of the primer. The dotted vertical lines represent the position of the AMV 4.5NF – AMDGR primer pair binding sites. [file Image_1.pdf]

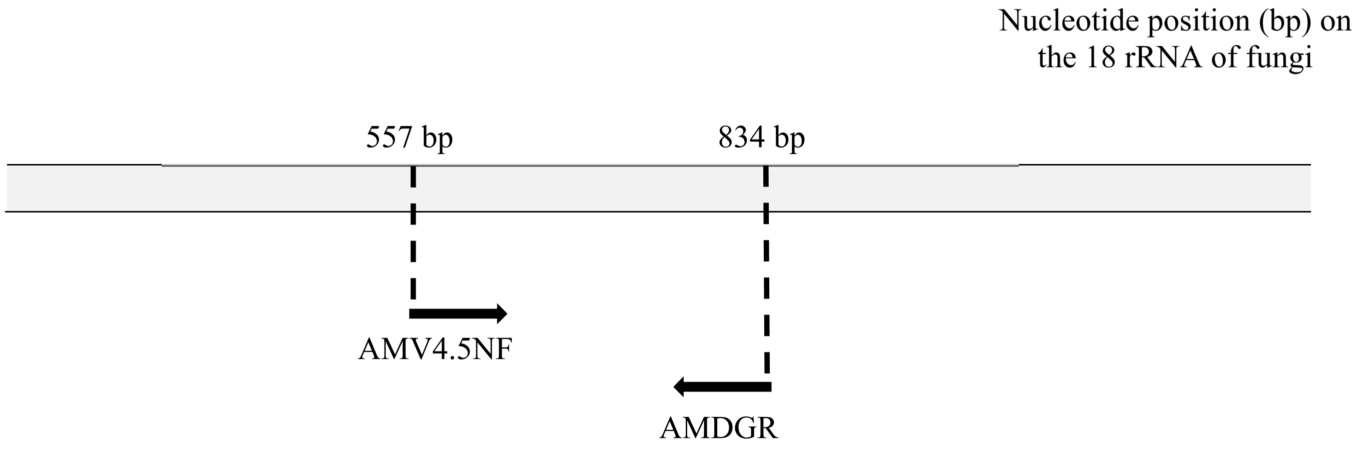

Supplementary Figure S 1. Position of binding of primer pair AMV4.5NF – AMDGR (Sato et al. 2005) on the small subunit of the 18S ribosomal RNA gene.
The lines represent the region by the specific primers. The arrows on the lines indicate the direction of sequencing of each primer. The dotted vertical lines represent the position of the AMV 4.5F – AMDGR primer pair binding sites.
