# Supplementary material for: Influence of plant species, mycorrhizal inoculant, and soil phosphorus level on arbuscular mycorrhizal communities in onion and carrot roots
Source: Front Plant Sci. 2024 Jan 15;14:1324626. doi: 10.3389/fpls.2023.1324626 (PMC10823018; doi:10.3389/fpls.2023.1324626)
Supplement: Supplementary Figure 2 — Relative abundance of arbuscular mycorrhizal fungal (AMF) genera colonizing the roots of onion and carrot plants grown in a muck soil in a field trial in Holland Marsh, Ontario, Canada. [file Image_2.pdf]

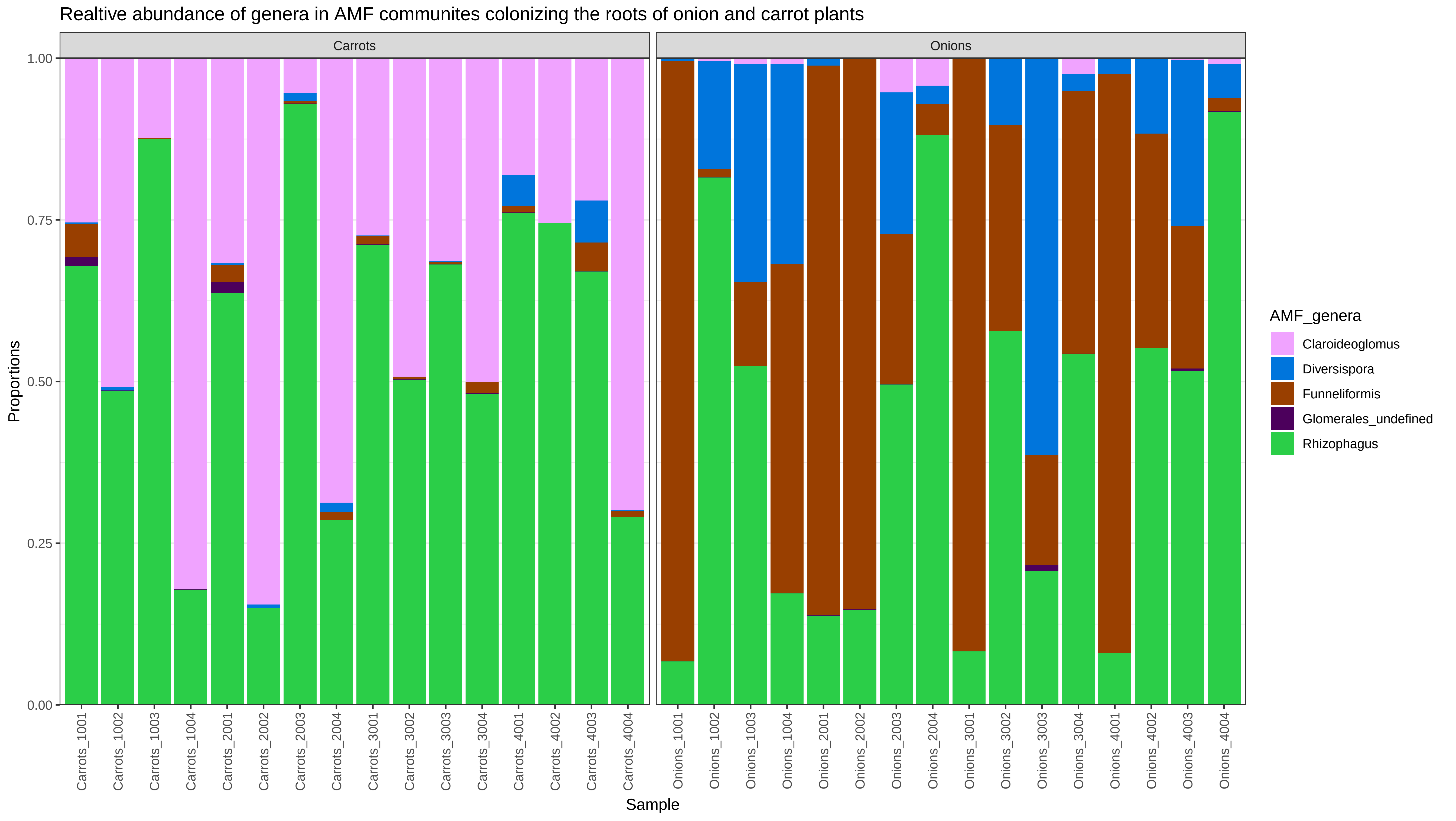

Realtive abundance of genera in AMF communites colonizing the roots of onion and carrot plants
Carrots
Onions
Proportions
0.00
0.25
0.50
0.75
1.00
Sample
Carrots_1001
Carrots_1002
Carrots_1003
Carrots_1004
Carrots_2001
Carrots_2002
Carrots_2003
Carrots_2004
Carrots_3001
Carrots_3002
Carrots_3003
Carrots_3004
Carrots_4001
Carrots_4002
Carrots_4003
Carrots_4004
Onions_1001
Onions_1002
Onions_1003
Onions_1004
Onions_2001
Onions_2002
Onions_2003
Onions_2004
Onions_3001
Onions_3002
Onions_3003
Onions_3004
Onions_4001
Onions_4002
Onions_4003
Onions_4004
AMF_genera
Claroideoglomus
Diversispora
Funneliformis
Glomerales_undefined
Rhizophagus
